# Can Forest Management Practices Counteract Species Loss Arising from Increasing European Demand for Forest Biomass under Climate Mitigation Scenarios?

**DOI:** 10.1021/acs.est.2c07867

**Published:** 2023-01-27

**Authors:** Francesca Rosa, Fulvio Di Fulvio, Pekka Lauri, Adam Felton, Nicklas Forsell, Stephan Pfister, Stefanie Hellweg

**Affiliations:** † Institute of Environmental Engineering, ETH Zurich, HPZ E33, John-von-Neumann-Weg 9, 8093Zurich, Switzerland; ‡ Ecosystems Services and Management Program (ESM), International Institute for Applied Systems Analysis (IIASA), Schlossplatz 1, A-2361Laxenburg, Austria; § Southern Swedish Forest Research Centre, Swedish University of Agricultural Sciences SLU, Sundsvägen 3, SE-230 53Alnarp, Sweden

**Keywords:** biodiversity, species richness, biodiversity footprint, life cycle thinking, bioeconomy, land use, leakage effects, closer-to-nature forests, set-aside, wood trade

## Abstract

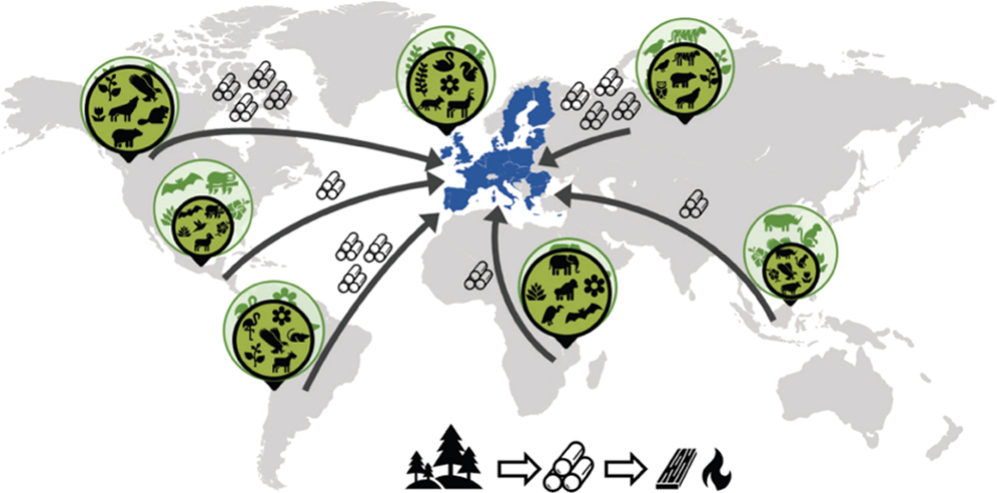

Forests are home to many species and provide biomass for material and energy. Here, we modeled the potential global species extinction risk from future scenarios of climate mitigation and EU28 forest management. We considered the continuation of current practices, the adoption of closer-to-nature management (low-intensity practices), and set-asides (conversion to unharvested forestland) on portions of EU28 forestland under two climate mitigation pathways as well as the consequences for the wood trade. Expanding set-aside to more than 25% of EU28 currently managed forestland by 2100 increased the global extinction risk compared to the continuation of current practices. This outcome stems from a projected increase in EU forest biomass imports, partially from biodiversity-vulnerable regions to compensate for a decrease in domestic harvest. Conversely, closer-to-nature management on up to 37.5% of EU28 forestland lowered extinction risks. Increasing the internal production and partially sourcing imported biomass from low-intensity managed areas lowered the species extinction footprint even further. However, low-intensity practices could not entirely compensate for the increased extinction risk under a high climate mitigation scenario with greater demand for lignocellulosic crops and energywood. When developing climate mitigation strategies, it is crucial to assess forest biomass supply chains for the early detection of extinction risks in non-EU regions and for developing strategies to prevent increase of global impacts.

## Abstract 2

While closer-to-nature management of EU28 forestland would have a lower biodiversity footprint compared to set-aside and the continuation of current practices, it does not compensate for the impacts caused by the increased demand for energy biomass under ambitious climate mitigation strategies.

## 1 Introduction

Human land use, especially by agriculture and forestry, is causing an unprecedented decline in global biodiversity.^1−3^ As forests host 70% of terrestrial species richness,^4^ the effective protection and sustainable management of forestlands is vital to stopping biodiversity loss. Achieving this will require integrated conservation policies.^5^ In Europe, where primary remaining forests are rare despite their conservation value,^6^ a new EU Biodiversity Strategy was approved in 2020. It was designed, along with a new EU Forest strategy in 2021,^7^ to lead biodiversity on the path to recovery by 2030.^8^ This is to be achieved via the increased protection and restoration of forest and nonforest habitats. These policies mainly focus on EU land, but they also mention the importance of assessing the potential impacts on non-EU biodiversity caused by resulting changes to wood imports. For example, increasing extensification of forestry within the EU to protect forest ecosystems would also lower wood yields and may increase wood imports to cover the demand. The implementation of strategies to protect biodiversity in the EU may thereby lead to a displacement of biodiversity impacts to regions outside the EU.^9^ In this regard, a new EU initiative was launched “to minimize the EU’s contribution to deforestation and forest degradation worldwide”^10^ as a follow-up to the communication on the EU’s contribution to the protection and restoration of the world’s forest.^11^ This is relevant to forest products as already in 2020, 20% of the EU28’s forest biomass demand for roundwood, semifinished products, and energywood was met by imports.^12^

The potential increase in EU28 forest biomass demand for material and energy use, especially under ambitious climate mitigation scenarios, could exacerbate the impacts of forest harvesting in non-EU biodiverse regions (hereafter termed “leakage”), with net negative impacts on global biodiversity.^13−16^ Such effects may be overlooked if climate mitigation strategies only focus on greenhouse gas emissions,^17^ especially considering that, so far, species extinction risk due to forest management has not been properly included in most policies.^4,18^ At the global scale, the assessments of future development scenarios up to 2050 compared to 2000 showed that in the absence of specific policies to reduce biodiversity loss, mean species abundance could decrease by 40% compared to the natural habitat,^19^ of which 5% due to forestry. Furthermore, forest cover changes and wood production could decrease vertebrate relative species richness by 12%.^20^ Moreover, regional studies on forest management have pointed out how the impacts on biodiversity can change considerably when different silvicultural approaches are applied^21,22^ and they have further shown that the benefits of either land sparing or land sharing is context-dependant.^4^

To our knowledge, an analysis of EU28 global wood supply-chain-related species extinction risk that clearly distinguishes between the relative contributions of various forest management intensities has not yet been performed. Moreover, published studies rarely assess EU28 forest management scenarios for long-term periods^23−26^ even though ecological effects due to habitat change are known to have significant temporal lags.^27^

In this paper, we addressed the following question: how do EU28 climate policies affect global species extinction risk up to 2100 and how could various changes in forest management mitigate or amplify these impacts?

Climate scenarios and wood demand predictions were modeled with the “Global Biosphere Management Model” (GLOBIOM)^28−30^ to develop future projections of land use and alternative forest management (AFM) scenarios in the EU28. EU28 local case studies performed in the H2020 ALTERFOR project^31,32^ were used to define the parameters of suitable forest management alternatives used in GLOBIOM. The land and forest use projections were combined with a life cycle impact assessment (LCIA) method that allowed a spatially explicit extinction risk of plants, mammals, and birds to be quantified (hereafter “global extinction risk”) from changes to both land use types and forest management intensity.^33−35^ This enabled us to assess the global extinction risk resulting from future EU28 demand for forest biomass and lignocellulosic energy crops, based on scenarios of climate mitigation and forest management practices. Thereby, we assessed the impact of the various forest management intensities adopted currently and in the projections^36^ as different management practices result in different productivity and impacts per managed forest area.^37−45^ We applied a long-term perspective, including projections up to 2100, and considered trade flows between economies in the global market to assess global impacts.^46−49^

## 2 Materials and Methods

Our methods consisted of two main parts (Figure S1.1 shows a flowchart of the whole procedure). First, we projected land use and forest management according to future scenarios (Section 2.1) using GLOBIOM^28−30^ (Section 2.2). GLOBIOM is an economic partial equilibrium optimization model of the global forest, agriculture, and bioenergy sectors with a bottom-up representation of agricultural and forestry management practices. Second, we modeled the global extinction risk, compared to a natural reference state, caused by projected land use with an improved version of an existing life cycle assessment (LCA) methodology^34,35^ (Section 2.3). This land-stress methodology^34,35^ was originally developed within the framework of life cycle assessment and is recommended as best practice by the United Nations Environmental Programme—Society of Environmental Toxicology and Chemistry (UNEP-SETAC) Life Cycle Initiative.^50^ We selected this methodology because it allowed us, with slight modifications to the method, to assess the impacts of various forest management intensities and nonmarginal changes.

### 2.1 Definition of Simulation Scenarios

The projections of future land use were modeled in GLOBIOM according to two climate scenarios within which multiple forest management scenarios were defined in terms of 10-year time steps (from 2020 to 2100).

As climate scenarios, we considered the Representative Concentration Pathways 6.5 and 2.6 (RCP6.5 and RCP2.6),^51^ modeled under the Shared Socioeconomic Pathway 2 (SSP2), an intermediate socioeconomic development scenario.^52^ RCP6.5^a^ and RCP2.6 are the no-mitigation (zero carbon price) and the high mitigation scenarios, respectively, leading to a 3.8 and 1.8 °C temperature increase in 2100 compared to preindustrial temperatures. The climate scenarios defined future EU28 demand for biomass energy production, sourced from EU28 forests andlignocellulosic energy crops and from energy plantations harvested in non-EU28 forests.

Within the framework of these two climate scenarios, ten different forest management scenarios for the EU28 were modeled using the results of the ALTERFOR project^31,32^ (see Section 2.2). These scenarios included different combinations of current forest management practices and alternative forest management practices (AFMs) adopted on different shares of European forestland (Table 1). The AFMs were grouped into two broad categories (Tables 1 and S2.1):

(1) Closer-to-nature forest management models (CFMs): AFMs closer to nature, with lower intensity than current forest management, such as selection (mature trees are selected for harvest to maintain closer to natural forest tree species and structural diversity), see Table S3.1 for more detailed definitions.
(2) Set-aside forest management models (SFMs): AFMs promoting set-asides, i.e., conversion of currently managed forest area to unharvested forestland instead of closer-to-nature forest management.

**Table 1:** Scenarios Used in the GLOBIOM Simulations from 2020 to 2100. Under the Forest Management Scenarios, the Area Converted to AFM Is 0 ha in 2020 and Reaches Its Maximum in 2100, According to the Given Share. AFM Denotes Both CFM and SFM, e.g., AFM25 Can Be CMF25 or SFM25.

scenario	description of the scenario	
**Climate Pathways**		
RCP6.5	representative concentration pathway 6.5 (+3.8 °C in 2100)	
RCP2.6	representative concentration pathway 2.6 (+1.8 °C in 2100)	
**EU28 Internal Forest Management**		
	Type of AFM	Area occupied by AFMs in 2100 (% of EU28 managed forestland in 2100)
noAFM	no alternative forest management (continuation of current practices)	0%
CFM12.5	closer-to-nature forest management	12.5%
CFM25	closer-to-nature forest management	25%
CFM37.5	closer-to-nature forest management	37.5%
CFM50	closer-to-nature forest management	50%
SFM12.5	set-aside	12.50%
SFM25	set-aside	25%
SFM37.5	set-aside	37.5%
SFM50	Set-aside	50%
**Supply-Chain and EU28 Managed Forest Area**		
Baseline	imported biomass from high-intensity forestry, according to historical trends.	
	total EU28 managed forestland in 2100 (excluding lignocellulosic energy crops): 134 Mha (equal to the current area under forest management).	
Shared-effort	11–29% of areas of wood supply outside the EU are low-intensity forestry.	
	total EU28 managed forestland in 2100 (excluding lignocellulosic energy crops): 160 Mha (increased compared to current area and equal to the total current EU forestland).	

The EU28 managed forest area not under AFM was projected to be managed according to more intensive practices, such as (in order of intensity level) (i) retention (single trees or group of trees are left in place to mitigate the effect of the harvest) and (ii) clear-cut (all of the trees of the areas are removed at once, resulting in even-aged silvicultures).

The parameterization of current forest management in GLOBIOM was adopted from the Global Forest Model (G4M) by Gusti et al.^53^. The results of the ALTERFOR project, which included data from nine case studies in eight European countries, provided the biophysical parameters used to model AFMs in GLOBIOM as changes relative to current management practices (Table S2.1).

As the case studies in ALTERFOR did not cover the entire EU28, the remaining areas suited to the application of AFMs were identified using a “suitability index”^54^ for 246 European administrative units (NUTS2). This index depended on climatic conditions, the proportion of conifer forest area, Shannon Diversity Index of tree species–area shares, and current management type. The increase or reduction of yield compared to the current state was also defined according to the similarity of a given area to the conditions found in the case studies.

The simulation of AFM implementation in GLOBIOM started in 2030 and ended in 2100, while in the period 2000–2020, there were no differences in the AFM scenarios. Table 1 describes all forest management scenarios, which included the continuation of current practices (no alternative forest management, noAFM), where AFMs were not implemented at all, as well as a set of options where CFMs or SFMs were implemented to different extents on EU28 forestland. Under the CFM and SFM scenarios, a linear expansion of the total area of AFMs from 2020 to 2100 was modeled (based on the final share in 2100). Economic optimization criteria determined the spatial allocation to areas deemed suitable for AFM. Each scenario also included the internal and import changes of biomass harvesting caused by shifts in forest management practice to land productivity within the EU28. A total area of 68 Mha, which corresponds to half of EU28 currently managed forestland (134 Mha), was deemed suitable for conversion to AFM. Initially, in the model it was assumed that wood imported for material and energy use came only from the most intensively managed forest areas, i.e., plantations for pulpwood and fuelwood and timber plantations, respectively (see Table S3.1). This assumption was based on economic criteria and historical trends.

Since initial results showed that the impacts of imports on global extinction risk played a bigger role than the impacts caused by EU28 domestic production, we performed an additional analysis. In this analysis, the EU28 forest area under management was extended to the whole EU28 forest area, 160 Mha (instead of the current 134 Mha) and the areas from where the imports came were partially set to low-intensity forest management (between 11 and 29%); in each climate and forest management scenario, this percentage depended on the economic competitiveness between the various forest management practices. The low-intensity forest practices considered for import were: (i) selection, in temperate and boreal regions and (ii) reduced impact logging, in tropical regions. This additional analysis is hereafter called “Shared-effort” as more effort is required by the EU28 to meet its internal demand by means of domestic production and to import biomass from sustainable practices as compared to the initial setting (hereafter called “Baseline”), where imports came only from high-intensity forestry and EU28 managed forestland remained constant at 134 Mha.

The yield considered in the modeling was specific to each forest management practice and country of origin (see Table S22).

### 2.2 Modeling of Land Use and Forest Management Development

The projections of future land use categories were performed with the GLOBIOM model^28−30^ at NUTS2 resolution within the EU28 and complemented by 29 GLOBIOM non-EU28 regions. The results were re-mapped at the ecoregion level^55^ to assess the extinction risk. For further details on the mapping of GLOBIOM regions, see SI, Section S4.

The model for land use change was built via two modules: a global module for all land uses (Section S5) and a module for the EU28 (hereafter EU-demand-module), the latter focusing on the fulfillment of the EU28’s demand for wood and lignocellulosic energy crops through domestic production within the EU28 and imports. EU28 production included roundwood and logging residues for material and energy use, as well as lignocellulosic energy crops. Additional roundwood, semifinished wood products, energywood, and wood pellets were imported from outside the EU28. The land use categories and forest management practices modeled in GLOBIOM were matched to the categories of the model to assess the extinction risk according to a correspondence matrix (Tables 2 and S5.1).

**Table 2:** Classification of Forest and Other Lignocellulosic Biomass Management Practices in GLOBIOM (Left Column) and the Corresponding Classification for the Response Ratios (RR) Available in the Model for the Extinction Risk Assessment (Right Column). The Symbols in Front of the Categories Distinguish between (i) Land Use Types in GLOBIOM Global Module (•) and EU-Deman-Module (◊) (Left Column) and (ii) Different Data Sources (†^34,35^, °^56^, ^■^(57,58)) (Right Column). A Double Symbol (•◊) Means That the Classification Applies to Both the Global and EU-Demand-Module.

GLOBIOM land use categories	response ratios^34,35,56^
•◊ lignocellulosic energy crops already used for energy production—internal EU28	† permanent crops
◊ lignocellulosic energy crops converted from other land use types—internal EU28	
	
◊ clear-cut—internal EU28 and imported into the EU28 through trade	° clear-cut
	
◊ retention system—internal EU28	° retention
	
◊ plantations for energywood already used for energy production—imported into the EU28 through trade	° timber plantation and plantation for pulpwood and fuelwood
◊ timber plantation and plantation for pulpwood—imported into the EU28 through trade	
◊ plantations for energywood converted from other land use types—imported into the EU28 through trade	
	
◊ selection system—internal EU28 and imported into the EU28 through trade	° selection system (temperate and boreal)
	
◊ reduced impact logging or RIL—imported into the EU28 through trade	° reduced impact logging or RIL (tropical)
	
• low-intensity managed forest	° merging of selective logging, selection system and retention
	
• high-intensity managed forest	° merging of clear-cut, timber plantations, nontimber plantations, and plantations for fuelwood and pulp
	
• forest regrowth	^■^ secondary forest
	the response ratios for this category were obtained by combining the results of a meta-analysis on biodiversity response in secondary forests^57^ and the modeling framework applied therein with the model defined in a more recent study on recovery trajectories^58^ (see S6 for details).

Although the EU-demand-module was nested in the global module and the climate scenarios, it was computed in GLOBIOM separately as two different models. Therefore, the results of the EU-demand-module were constrained to stay within the land use boundaries of the global module in each GLOBIOM region, while providing a more refined downscaling of the forest management practices. As a result, the projections from the two modules were integrated into a land use matrix that was able to combine changes in the global module and the EU-demand-module (Section S7).

The EU-demand-module of GLOBIOM considered different forest management scenarios implemented in the EU28 and subsequent EU28 internal changes (resolution of NUTS2), as well as changes in areas outside of the EU28 where the biomass was harvested and imported to the EU28 to meet internal wood demand (resolution of GLOBIOM regions). Forestland was classified as either (i) “primary” (no exploitation), (ii) “regrowth” (past but not current exploitation), or (iii) “managed” (current active exploitation). Additionally, the area supplying wood to satisfy EU28 wood demand (internally and through wood imports), and falling within the “managed forest” category, was classified according to management intensities. The forest management practices considered in the EU28 for AFMs were “selection” and “retention”, while the remaining areas were covered with “clear-cut”. The initial area under management per EU28 country was calibrated to match the total area under management and harvested wood volumes from the FAO.^12,59^ A cost function for management changes regulated the transition between different management practices. These transitions were controlled by the mapping of permitted management changes and of areas suitable for alternative management practices in the EU.^54^ The forest sector was represented by modeling in GLOBIOM the forestry subsector, the forest industry subsector, and the bioenergy subsector, as described in Lauri et al.^60^ Managed forest areas not explicitly modeled in the EU-demand-module (hereafter “other management”) were defined according to a coarser split of management between “high-intensity managed forests” and “low-intensity managed forests” according to FAO data,^12^ after calibration.

The EU-demand-module also calculated the extent of areas converted to lignocellulosic energy crops (within the EU28) and energy plantations (outside the EU28) from other land uses (cropland, grassland, other natural vegetation) to satisfy EU28 demand, which was not considered in the AFMs.

### 2.3 Modeling of Extinction Risk Due to Land Use

Extinction risk due to land use was assessed by improving and adapting the LC-Impact methodology for land-use stress^34,35^ to the present study. The methodology is based on the countryside species–area relationship (countryside-SARs):^34,61^
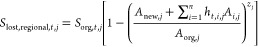
1where *S*_lost,regional,*t*,*j*_ is the number of species lost in ecoregion *j* for species group *t*. It corresponds to the difference between the original number of species in undisturbed habitat in ecoregion *j* (*S*_org,*t*,*j*_)^34^ and the number of species occurring in human-modified land use. *A*_new,*j*_ is the remaining natural habitat area in ecoregion.^28−30^*A*_*i*,*j*_ is the area covered by land use type *i* in ecoregion *j*.^28−30,62,63^*A*_org,*j*_ is the original natural habitat area in ecoregion *j*(28−30)*. h*_*t*,*i*,*j*_ is the affinity of species group *t* to land use category *i* in ecoregion *j*, calculated as
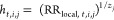
where RR is the response ratio *S*_*t*,*i*,*j*_/*S*_org,*t*,*j*_, i.e., the ratio between the number of species belonging to species group *t* and occurring in land use type *i* in ecoregion *j* and the number of species belonging to species group *t* occurring in the natural habitat of ecoregion *j*.^34,56^*z*_*j*_ is the slope parameter of SAR for ecoregion *j* and, in this model, depends on the habitat type to which the ecoregion belongs (forest, nonforest, island).^64^ Species loss at the ecoregion scale (eq 1) was converted to extinction risk using species vulnerability scores (0 ≤ VS ≤ 1). VS are a function of range area and red-list threat level (IUCN) and are a proxy for the probability that a regional species loss results in the global extinction of the species. The final results were expressed as global Potentially Disappeared Fraction of species (PDF), which indicates the fraction of species at risk of becoming globally extinct in each scenario. In contrast to the original methodology,^34^ we entered the GLOBIOM land use areas in the first equation of the model (eq 1) and computed the impacts accordingly for each scenario. This approach prevented linearity assumptions—usually applied in LCA but not representative of the underlying ecological model, especially when assessing large land use occupation—for the exponential relationship that characterizes the countryside SAR. For detailed calculations, see the original publicationpublications^34,35^ and Section S8.

The following two major adjustments were made to adapt the methodology to our focus on forest management and to take related uncertainties into consideration.

(1) An extra subclassification of forest management intensities was added. The original method^34,35^ considers six land use types: annual and permanent crops, pastures, urban, and high- and low-intensity forests. The latter two were further classified into five subclasses of forest use intensities (Tables 2 and S5.1) via the inclusion of additional data providing the response of species richness to different forest management alternatives.^56^ We therefore derived forestry-intensity-specific affinity values for different species groups (see S9). The forest use management practices projected by the land use model could thereby be linked to the most fitting forest use intensities of the habitat used to quantify the extinction risk.
(2) For an estimation of uncertainties, we applied bootstrapping to the response ratios and the *z* values, which were the most relevant contributors to the variance of eq 1,^34^ and for which the whole set of raw values was available^34,56,64^ (see S10). We could therefore quantify the confidence intervals of the expected values obtained entering the median of RR per land use, species group and ecoregion, and the median of *z* per habitat type and species group in eq 1 (see S9 and S10). We chose this approach as it was suited to the limited data availability of RR for some combinations of land use, species group, and ecoregions (the data merging procedure we applied if less than five data points were available for a given combination is described in S9).

The assessment was only performed for plants, mammals, and birds because there was not enough data on the response ratios of amphibians and reptiles for the different forest management intensities. We do not expect this limitation to critically impact the calculation as a high correlation was found between the characterization factors (CF) originally developed for the LC-Impact methodology^34^ and the CF obtained aggregating the three species groups selected for this study (S23). Characterization factors are multiplicative factors used in the life cycle assessment to convert the environmental pressures into impacts. In this study, we gave the same weight to all species groups when we aggregated the results.

The model used to compute the extinction risk and all of the allocation processes were built in R4.0.^65^

### 2.4 Sensitivity Analysis

A sensitivity analysis (see Section S12) was conducted to investigate two aspects. (i) How impacts changed when converting a share of clear-cut areas in the EU28 to timber plantations (a more intensive forest management practice characterized by less diversity in species). This analysis was performed, because in the GLOBIOM model, some areas in the EU28 classified as clear-cut were described as managed with practices potentially more intensive than standard clear-cut practices (plantations of monospecific non-native species, although not as intensive as on conventional tropical timber plantations, which have a high frequency of harvesting and include the application of pesticides and fertilizers). (ii) An extreme forest management scenario (hereafter *laissez-faire*) where the GLOBIOM model was left free to choose the most economically convenient options for EU28 internal forest management, with very few constraints. This scenario projected the harvesting of wood almost exclusively from intensively managed forestland in the EU28. We tested this option to see how the extinction risk would react to a strategy that increases the intensity of EU forest management for internal consumption (opposite direction compared to the other scenarios).

## 3 Results

The following results refer to the Baseline scenario (EU28 managed forestland on 134 Mha and imports only from intensive forestry, see Table 1) unless the Shared-effort scenario is explicitly referred to (EU28 managed forestland on 160 Mha and imports partially from low-intensity forestry).

### 3.1 EU28 Domestic Impacts and Footprint

**Figure 1:**
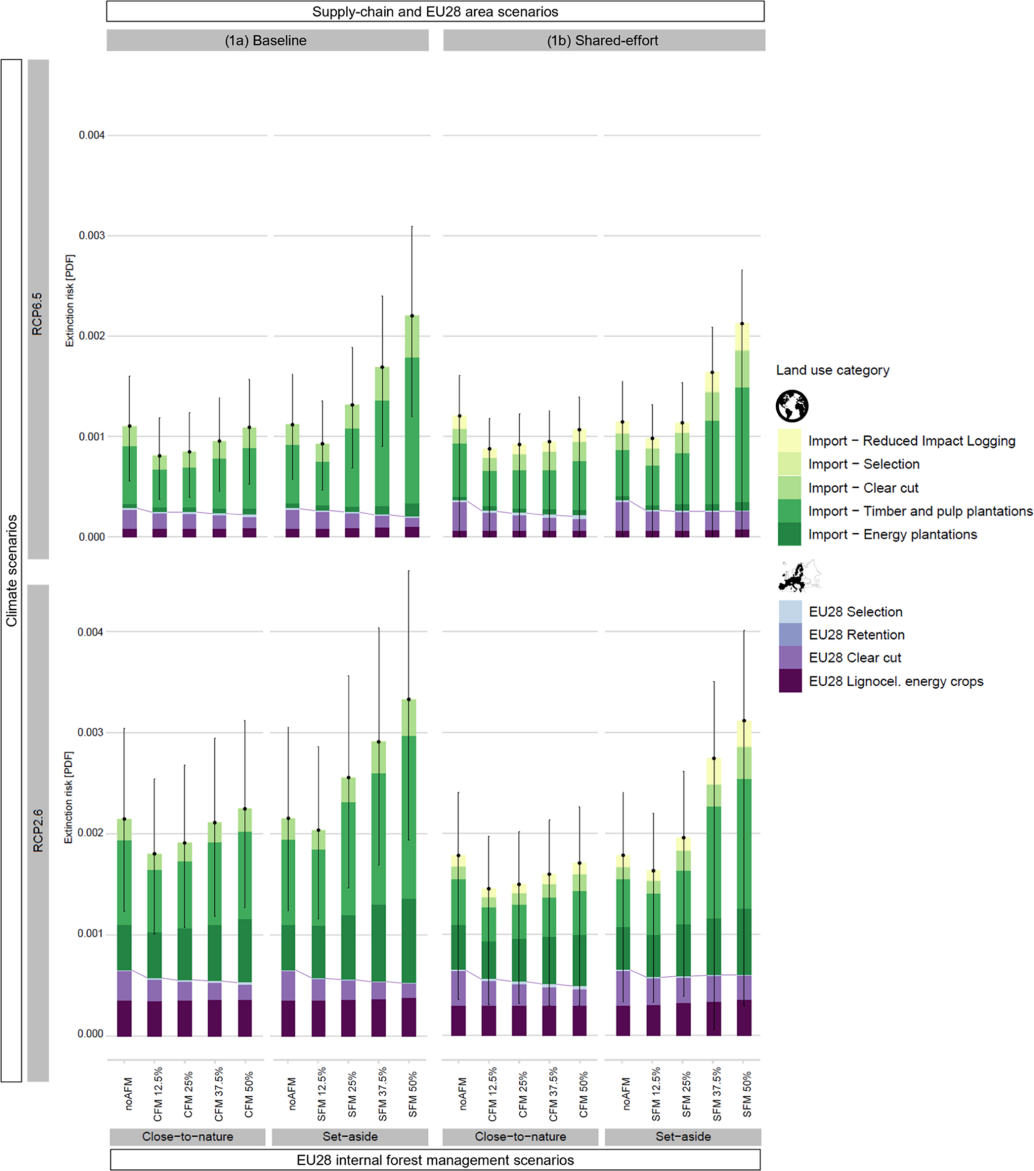
Extinction risk in 2100 due to demand for EU28 forest biomass and lignocellulosic energy crops under different climate and forest management scenarios. The stacked bars display the contributions of different forest management practices, while the error bars indicate the 95% confidence intervals of the cumulated impact. For the sake of clarity, purple lines between the bars have been added to show the EU28 internal impacts. noAFM = no alternative forest management, CFM or SFM12.5%/25%/37.5%/50% = closer-to-nature management or set-aside adopted on 12.5%/25%/37.5%/50% of EU28 managed forestland. Lignocel. = Lignocellulosic.

Concerning the domestic impacts on the extinction risk within the EU28 (without considering the impacts from wood imports or exports), in 2100, the extinction risk slightly decreased in most cases with the increase of closer-to-nature and set-aside implementation compared to noAFM (Figure 1a, blue-violet part). The lowest extinction risk caused by internal EU28 forestry was observed for CFM50 and SFM50 scenarios, when 50% of currently managed forestland (68 Mha) was converted either to closer-to-nature or set-aside practices (Figures 1a, S13a, and S24). The extinction risk in CFM50 and SFM50 was 15–22% smaller than noAFM. The increased demand for energy biomass under the most ambitious climate mitigation pathway played a big role (Figures 1a and S13a, violet part): for the same AFM scenarios, the impacts under RCP2.6 were estimated to be almost twice those of RCP6.5.

When we broaden our consideration to include a demand perspective, the impacts of biomass imports must be also included, as EU28 internal production is insufficient to meet the domestic demand for woody biomass as material (roundwood, semifinished products) or energy use (wood pellets, logging residues, lignocellulosic crops). In 2100, under the RCP6.5 climate scenario, the demand for roundwood equivalent coming both from forestry and lignocellulosic crops was projected to be 650 Mm^3^, of which 41% in CFM50 and 67% in SFM50 was met by imports (Figure S13a). Under RCP2.6, the demand reached 1600 Mm^3^ of roundwood equivalent, of which imports covered 43% in CFM50 and 63% in SFM50 (Figure S13a). Under RCP2.6, the demand reached 1600 Mm^3^ of roundwood equivalent, of which imports covered 43% in CFM50 and 63% in SFM50 (Figure S13a). As a consequence, the extinction risk under the RCP2.6 was projected to be 1.5–2.1 times larger than under the RCP6.5 due to the significantly higher demand for wood and lignocellulosic energy crops.

Over time, the extinction risk directly associated with EU28 woody and lignocellulosic biomass demand notably increased in all scenarios (Figure S14.2), with the maximum extinction risk in 2100 for both domestic impacts and impacts outside the EU.

The EU28 global footprint (Figure 1a) showed a very different trend compared to EU28 internal impacts (cf. Figure 1a, blue-purple part). While EU28 internal impacts decreased with reduced intensity, the adoption of closer-to-nature and set-aside practices on limited areas caused a smaller global extinction risk than their adoption over large areas (due to the increasing impacts of imports).

The extinction risk under the SFM12.5 scenarios was lower than noAFM, whereas all scenarios with higher implementation of SFM (SFM25–50) exceeded the noAFM due to greatly increased imports to meet EU28 demand, especially in the high climate mitigation scenario. For example, under the RCP2.6, imported wood amounts in SFM50 were 474 Mm^3^ of roundwood equivalents higher than in noAFM (1001 vs 527 Mm^3^), which resulted in a +50% extinction risk compared to noAFM.

In the CFM scenarios, the rate of increase in impacts associated with the amount of AFM area was less steep than in the SFM scenarios and the extinction risk remained below that of noAFM, except for CFM50 under RCP2.6.

When imported wood was partially sourced from low-intensity forestry and EU28 managed forestland was extended to 160 Mha (Shared-effort scenario, see Figure S13b), the extinction risk in most climate and forest scenarios was lower than the corresponding Baseline scenarios, especially under CFMs and RCP2.6 (see Figure 1a,b). The impacts under RCP2.6 CFM37.5, CFM50, and SFM25 were 24% lower than in the comparable Baseline scenarios and none of the RCP2.6 CFM scenarios exceeded the noAFM. Only for the RCP6.5 CFM12.5, SFM12.5, and CFM25 scenarios did the Shared-effort scenario have 5–8% larger impacts than the corresponding Baseline scenarios.

For high volumes of imported biomass (namely, under the RCP2.6 scenario), the impacts per Mm^3^ of roundwood equivalent of imported wood (excluding energy plantations) were up to 26% lower in the Shared-effort than in the Baseline (Table S15.1). Conversely, they were higher in most scenarios under RCP6.5. A similar trend characterized the extinction risk per imported Mm^3^ for energy plantations increased (Table S15.1).

Concerning the impacts per species group (see S16), the highest contribution to extinction risk came from declines in plant and bird species (Figures S16.1 and S16.2). Mammals seemed less sensitive to intense forest management (Figures S16.1c and S16.2c), as supported by several studies.^66−68^

The sensitivity analysis showed that the expansion of timber plantations in the EU28 onto a subset of clear-cut forestland did not affect the global footprint significantly, although domestic EU28 impacts increased up to 9% compared to those scenarios where timber plantations were excluded (Figure S12.1).

The results of the analysis of the *laissez-faire* forest management scenario (S12) indicated that EU28 internal impacts would be smaller than noAFM but similar to or higher than the CFM and SFM scenarios, while the global extinction risk (also considering imports) would be smaller than all of the other scenarios.

### 3.2 Contribution of Different Forest Management Practices

Given the context of the study, it is important to consider the contribution of the various forest management practices in terms of biomass harvested, areas needed, and impacts caused.

In both climate scenarios, intensive systems covered most of the demand but had high overall impacts despite occupying a limited area. In contrast, low-intensity forestry occupied a substantial area but caused relatively small impacts.

For example, under RCP6.5 and RCP2.6 CFM50, 12% (109 Mm^3^ of 883 Mm^3^) and 28% (435 Mm^3^ of 1564 Mm^3^), respectively, of the biomass harvested came from timber, pulp, and energy plantations (outside the EU28), which occupied only 7% (15 Mha of 204 Mha) and 13% (31 Mha of 247 Mha) of the area used to meet EU demand but caused 61 and 66% of the impacts. On the other hand, in the same scenarios, 34% (304 Mm^3^) and 21% (340 Mm^3^) of the biomass harvested came from EU28 selection forestry, which occupied 38% (78 Mha) and 28% (70 Mha) of the area but caused almost negligible impacts. Concerning low-intensity forestry outside the EU, in the Shared-effort option, and under the same climate and AFMs scenarios mentioned above, the imported biomass harvested from selection and reduced impact logging was 4% and 2% of the total biomass amount harvested, while occupying 16% and 14% of the area used to meet EU28 demand (Figure S13b) and having 11% and 6% of the impacts. The impacts mostly came from reduced impact logging, which is usually implemented in tropical areas (Figure 1b). See S22 for different productivity values of the various forest management practices.

### 3.3 Spatial Distribution of the Impacts

In 2020, according to the GLOBIOM model calibration, 14% of the land needed to produce wood products was sourced from tropical regions. This percentage increased to 17–22% by the year 2100 under the different AFM scenarios. Even if tropical and subtropical regions made a limited contribution in terms of the amount of wood imported, they host the most vulnerable species and suffer from the most severe extinction risk from biomass production.

**Figure 2:**
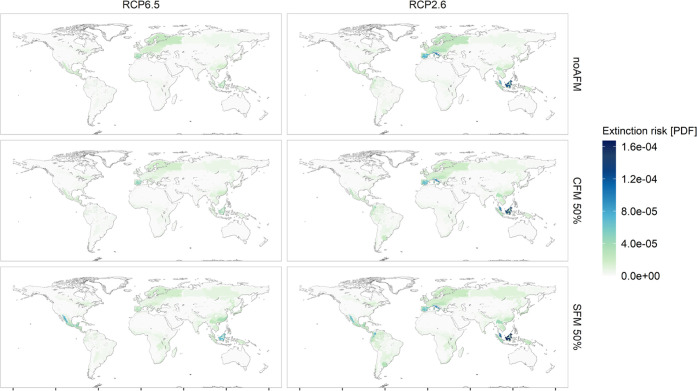
Spatial distribution of global extinction risk in 2100 caused by demand for EU28 wood and lignocellulosic energy crops at ecoregion resolution under the two climate scenarios RCP6.5 and RCP2.6 and the most extreme alternative forest management scenarios, where half of EU28 forestland currently under forest management is converted to closer-to-nature practices or to set-asides.

A comparison between climate scenarios and forest management scenarios (Figures 2 and S17.1) in 2100 shows, on the one hand, the remarkable shift of regional impacts to Southeast Asia when comparing RCP2.6 to RCP6.5; on the other hand, the more closer-to-nature and set-aside approaches are adopted in the EU, the more regional impacts were shifted to Latin America. The PDF per Mm^3^ of roundwood equivalent imported from non-EU28 forestland in 2100 was an order of magnitude higher than from EU28 forests (Table S18.1, Figures S18.1, and S18.2). Increased imports from locations rich in endemic species, such as the tropics, therefore caused a considerable increase in extinction risk.

The management intensity level outside of the EU28 did not influence the spatial distribution of the most impacted regions (Figure S20.1) in spite of the differences in the absolute magnitude of impacts (Figure 1).

## 4 Discussion

In this study, we conducted a spatially explicit assessment of the global extinction risk for plants, mammals, and birds until 2100 under multiple scenarios of EU28 climate mitigation and forest management, while taking into consideration EU28 wood and energy crops biomass demand. The implementation of closer-to-nature forest management reduced EU28 internal extinction risk and the global footprint compared to noAFM. The use of selection (see Section 3.2) would allow the EU28 to cover part of its wood demand with internal low-impact forestry practices, without substantially increasing the need for imports. However, the benefits achieved would not be enough to fully compensate for increasing global extinction risk that results from the high demand for biomass for energy production that occur under the RCP2.6 high mitigation climate scenario.

In contrast to closer-to-nature forest management, the global extinction risk caused by set-aside practice in the EU28 was larger than noAFM, even when set-aside was only implemented on 25% of EU28 currently managed forestland. This is because of the high increase in imports that was needed to meet EU28 biomass demand: the EU28 internal benefits of set-aside were nullified by the leakage of biodiversity impacts outside the EU28.

The replacement of clear-cut and plantations with selection and reduced impact logging in the regions outside of the EU28 on up to 29% of the areas together with increased internal production (Shared-effort scenario) reduced the overall extinction risk in most scenarios (see Figure S13b). Despite the lower overall extinction risk, the imports coming from those regions where high-intensity management was still in place, such as plantations, still caused high impacts in the Tropics. The importance of the geographical provenience of the biomass imports was also relevant when different low-intensity management practices implemented outside the EU28 were compared (namely, selection and reduced impact logging): despite covering overall the same or even a lower area, reduced impact logging caused more impacts than selection because the former is common practice in the Tropics (where many endemic species live) while the latter is generally applied in temperate regions (Section 3.2).

These results underlined the importance of (i) applying a footprint perspective to prevent outsourcing extinction risks, especially when new land use strategies or ambitious climate change policies are adopted; (ii) distinguishing various levels of forest management intensity (using a finer resolution than simply “high-intensity” and “low-intensity” forest management); and (iii) identifying areas undergoing land use or management change due to wood trade to preempt and thereby possibly prevent the expansion of forest management into regions highly vulnerable to extensive biodiversity loss.

Concerning the results of the *laissez-faire* scenario in the sensitivity analysis, it is important to mention that, despite the results obtained, it is highly unlikely that this scenario would be implemented given that it implies the adoption of an intensity of forest management that is in direct conflict with EU28 environmental policies.

### 4.1 Strengths and Limitations

The uniqueness of our study stemmed from considering the effect of forest management on biomass yield as well as on the global extinction risk of plants, mammals, and birds. For this, local case studies were upscaled to the European scale and global trade implications were modeled. The evaluation of extinction risk was performed for specific forest management practices and took nonlinear ecological models into account. Compared to Di Fulvio et al.,^69^ we were thereby able to obtain more consistent modeling outcomes over time for the implementation of different AFMs, and we had a more detailed representation of forest management. Therefore, the leakage effects due to imports could be assessed with higher accuracy.

The countryside SAR model coupled with GLOBIOM areas allowed us to quantify potential extinction risk at the global scale, focusing on forest management and climate change mitigation scenarios. The distinction among management intensities and spatial specificity (e.g., selection systems in temperate areas versus reduced impact logging in tropical areas) proved highly important to result outcomes. Moreover, a more robust uncertainty assessment compared to previous studies was provided. Regarding result uncertainty, bootstrapping allowed us to set confidence intervals for the expected values of the extinction risk model. Our estimated impacts on extinction risk relied on multiple factors and, especially for the response ratios, data limitations and assumptions significantly contributed to the uncertainty of the results.

Finally, the data source used for the additional response ratios of the different forest management intensities proved to be the most appropriate for the current study as its mode of classification for management intensities matched those used in GLOBIOM and had a suitable level of granularity, which was not the case for other published meta-analyses or databases.

To further develop the methodology, it would be useful to include more detailed biodiversity metrics capable of capturing differences between a larger set of forestry management practices, as done at the local scale by Rossi et al.,^22^ as well as to improve the robustness of the factors used to scale the extinction risk from regional to global levels.^70^ Additionally, refining the spatial allocation of current forest management practices could help to better calibrate the GLOBIOM model in Europe.^71^ Furthermore, additional scenarios of global forest management practices outside the EU28 could be added.

The following aspects were not addressed in this work due to a lack of data or owing to model limitations, but could be beneficial for application in future studies: (1) the impact of land fragmentation,^72^ (2) impacts on species composition or functionality,^73,74^ and (3) differentiating impacts among tree species since different tree species may provide complementary benefits for biodiversity.^43,75^

Our analyses were limited to lands used directly to supply forest industries and lignocellulosic energy crops. Although shifts in land use from the EU28 to exporting countries were considered, we did not take into account additional indirect land use changes. For example, forest commodities may cause an increase in deforestation^29^ even if ca. 90% of deforestation through global trade is attributed to agricultural and not forest commodities.^48^ Similarly, a steady conversion of agricultural and natural land to energy production areas under RCP2.6 could cause food shortages, agricultural intensification, and indirect deforestation, generating a trade-off between food security, conservation, and climate mitigation.^76−78^ However, this was not modeled (see Table S21.1 for the areas converted by the GLOBIOM model from croplands, grasslands, and natural lands to lignocellulosic energy crops and energy plantations, which would thereby need to be displaced). Extreme climate scenarios could cause similar effects in the absence of mitigation strategies.^79,80^

We acknowledge that assessing outcomes in the year 2100 comes with many uncertainties in terms of development pathways and impacts. However, it is important to analyze the potential outcomes of different development trajectories and land use strategies in the long term to be able to undertake course correction and act accordingly.^5^

### 4.2 Policy Recommendations

As stated in the new EU initiative, to prevent worldwide deforestation and forest degradation it is essential to establish forest-friendly trade, and this principle equally applies to the development of climate change mitigation strategies. To avoid or limit global extinction risk, the EU28 could turn to additional mechanisms that encourage sustainable forestry practices by promoting, for example, environmental certification schemes. For example, trade policy interventions prioritizing wood imports from boreal and temperate regions would reduce the EU28 biodiversity footprint from wood imports^81^ and result in more favorable outcomes for biodiversity than those projected in this study. Another consideration arises from the best use of wood to reduce climate change. For example, other studies have shown that the use of wood biomass in long-lived products (e.g., replacing energy-intensive materials in the construction industry) in combination with their potential cascade use and a final end-of-life energetic valorization is crucial to increasing the climate mitigation potential of wood biomass via biogenic carbon flows.^82^

Furthermore, our results suggest that when planning climate change mitigation policies, it is crucial to define land-management strategies using both a regional perspective to preserve local biodiversity while simultaneously considering the potential global leakage of biodiversity impacts. This implies that the geographic origin of woody biomass and the management system to harvest must always be considered to avoid a shift of extinction risk to highly vulnerable regions. Implementing closer-to-nature forest management in a higher percentage of forest area in the EU28 can potentially mitigate some of the impacts resulting from climate-ambitious scenarios. By contrast, expanding set-aside forest areas over a certain threshold in the EU28 would potentially create leakages outside the region and, overall, result in a net increase in global species extinction risk. Therefore, it is imperative for EU policy makers to identify forest management pathways that help to mitigate both climate change and the unsustainable loss of global biodiversity both within and outside the EU. We thus recommend that integrated climate-biodiversity scenarios and policies be defined.

## Data Availability

The code required to run the analyses presented here can be obtained from the GitHub repository at https://github.com/francesca-git/EU28-ForestMng-Climate.

## Supporting Information Available

Methodology flowchart; parameters used for alternative forest management (AFM) representation in GLOBIOM; definitions of forest management practices; mapping of regions in the projections of land use; GLOBIOM model for the projections of the global land use module; response ratios for secondary forests; allocation of land use areas; modeling of species loss–methodological detail; raw data used in the calculation of the response ratios and the *z* values; bootstrapping and propagation of uncertainties; map of ecoregions included and excluded from the study; sensitivity analysis; biomass harvested and areas; development over time of the species extinction risk; impacts per unit of imported volume; spatial distribution of impacts for the different species groups; spatial distribution of the species extinction risk; impacts on the species extinction risk per unit of volume for harvested forest product; difference and ratio compared to noAFM; spatial distribution of impacts in the Shared-effort scenario; and areas converted to lignocellulosic energy crops and energy plantations (S1–S21) (PDF)
Yield of the various forest management practices considered, according to their geographical origin and whether they are subject to alternative forest management (AFM) or not (S22) (XLSX)
Correlation analysis between characterization factors obtained with five or three species groups (S23) (XLSX)
Absolute values of the extinction risk assessment (S24) (XLSX)

## Abbreviations Used

RCP6.5: representative concentration pathway leading to a 3.8 °C temperature increase in 2100 compared to the preindustrial level
RCP2.6: representative concentration pathway leading to a 1.8 °C temperature increase in 2100 compared to the preindustrial level
SSP2: middle of the road shared socioeconomic pathway
AFM: alternative forest management
AFM12.5/AFM25/AFM37.5/AFM50: Alternative Forest Management adopted on 12.5%/25%/37.5%/50% of EU28 managed forestland in 2100
CFM: closer-to-nature forest management
CFM12.5/CFM25/CFM37.5/CFM50: Close-to-nature Forest Management adopted on 12.5%/25%/37.5%/50% of EU28 managed forestland in 2100
SFM: set-aside forest management
SFM12.5/SFM25/SFM37.5/SFM50: Set-aside Forest Management adopted on 12.5%/25%/37.5%/50% of EU28 managed forestland in 2100
*laissez-faire*: scenario free to choose among alternative forest management and high-yield forest management practices according to economic criteria
LCA: life cycle assessment
GLOBIOM: global biosphere management model
NUTS: nomenclature of territorial units for statistics (French: nomenclature des unités territoriales statistiques)
RR: response ratio
SAR: species–area relationship
PDF: potentially disappeared fraction of species
VS: vulnerability scores

## Footnotes

a The RCP6.5 scenario was selected as the no-mitigation scenario as it is compatible with the SSP2.
