# Supplementary material for: Can Forest Management Practices Counteract Species Loss Arising from Increasing European Demand for Forest Biomass under Climate Mitigation Scenarios?
Source: Environ Sci Technol. 2023 Jan 27;57(5):2149–61. doi: 10.1021/acs.est.2c07867 (PMC9910049; doi:10.1021/acs.est.2c07867)
Supplement: Supplementary file 1 — es2c07867_si_001.pdf [file es2c07867_si_001.pdf]

Supporting Information S1 – S21

Can forest management practices counteract species loss arising from increasing European demand for forest biomass under climate mitigation scenarios?

Francesca Rosa[1], Fulvio Di Fulvio[2], Pekka Lauri[2], Adam Felton[3], Nicklas Forsell[2], Stephan Pfister[1], Stefanie Hellweg[1]*

* Email: rosa@ifu.baug.ethz.ch

[1]Institute of Environmental Engineering, ETH Zurich, HPZ E33, John-von-Neumann-Weg 9, 8093 Zurich, Switzerland

[2]Ecosystems Services and Management Program (ESM), International Institute for Applied Systems Analysis (IIASA), Schlossplatz 1, A-2361 Laxenburg, Austria

[3]Southern Swedish Forest Research Centre, Swedish University of Agricultural Sciences SLU, Sundsvägen 3, SE-230 53 Alnarp, Sweden

Summary:

21 sections, 52 pages, 26 figures, 8 tables.

Table of Contents

S1	Methodology flowchart	3
S2	Parameters used for Alternative Forest Managements (AFM) representation in GLOBIOM	4
S3	Definitions of forest management practices	6
S4	Mapping of regions in the projections of land use	7
S5	GLOBIOM model for the projections of the global land use module	12
S6	Response ratios for secondary forests	15
S7	Allocation of land use areas	17
S8	Modelling of species loss – methodological detail	20
S9	Raw data used in the calculation of the response ratios and the z values	25
S10	Bootstrapping and propagation of uncertainties	28
S11	Map of ecoregions included and excluded from the study	30
S12	Sensitivity Analysis	31
S13	Biomass harvested and areas	35
S14	Development over time of the species extinction risk	36
S15	Impacts per unit of imported volume	38
S16	Spatial distribution of impacts for the different species groups	39
S17	Spatial distribution of the species extinction risk	41
S18	Impacts on the species extinction risk per unit of volume for harvested forest product	42
S19	Difference and ratio compared to noAFM	46
S20	Spatial distribution of impacts in the Shared-effort scenario	48
S21	Areas converted to lignocellulosic energy crops and energy plantations	49

S1 Methodology flowchart

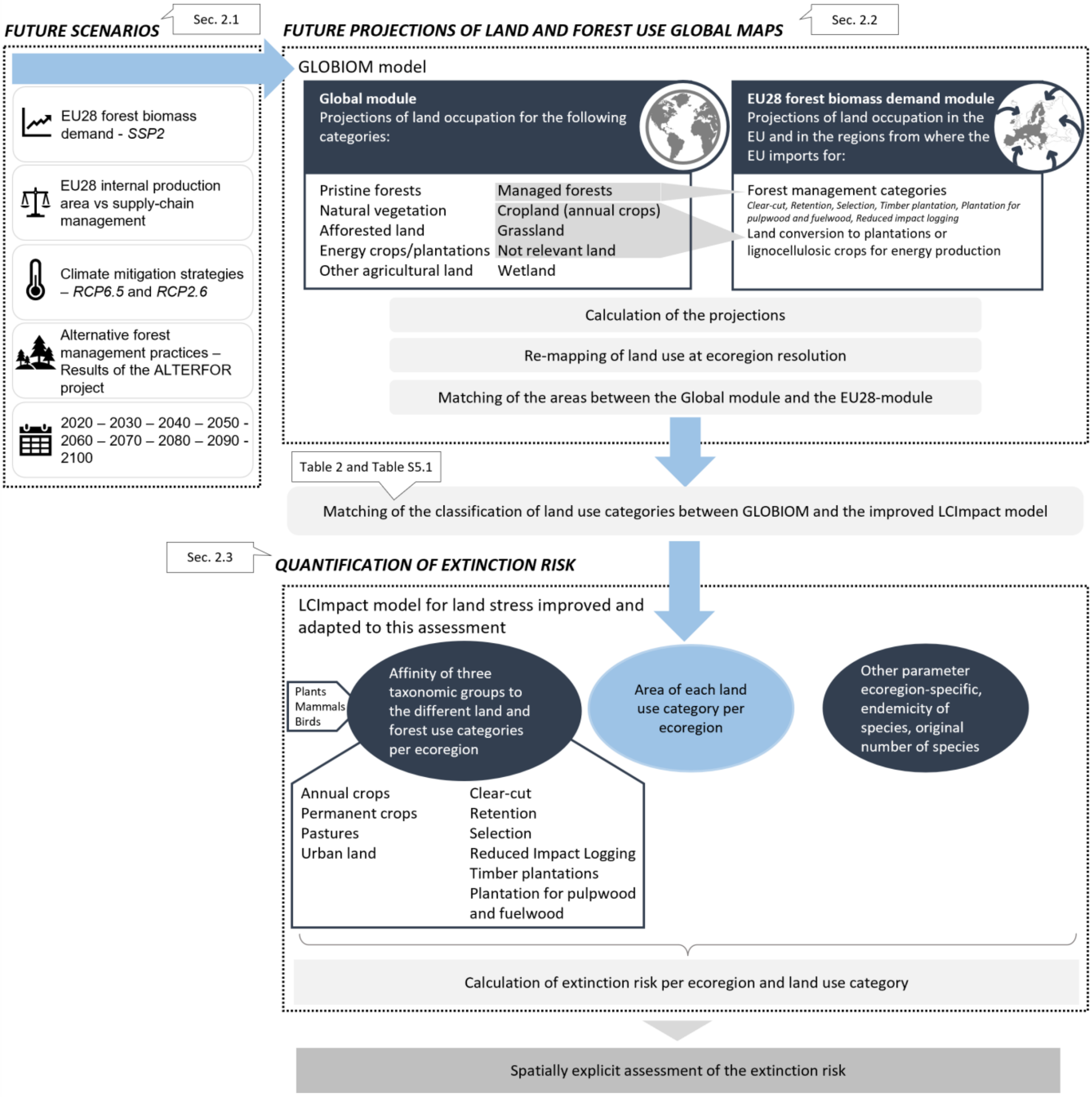

Figure S 1.1 *Flowchart of methodology used to quantify species loss.*

S2 Parameters used for Alternative Forest Managements (AFM) representation in GLOBIOM

FM: Forest management

AFM: Alternative Forest Managements

CFM: Close-to-nature management.

SFM: Set aside (un-management of forest area currently under forest management).

PFM: Production forest management.

Table S2.1 contains the parameters which resulted from the EU28 case studies of the ALTERFOR project, and which were used in the GLOBIOM model to define the area suitable for the AFMs.

Table S 2.1 *Details on the representation of AFMs in GLOBIOM as a result of the ALTERFOR project.*

Management name	*Type of management*	*Harvested increment (ratio to current FM)*	*Non-coniferous share relative to the current representative management withing the same region.*	*Suitable area in the EU28 (Mha)*
CFM_DE	*Selection system*	0.93	1.56[1]	15.1
PFM_DE	*Retention*	1.18	0.58	15.1
SFM_DE	*Set Aside*	0.00	NA[2]	15.1
CFM_IT_1	*Selection system*	0.86	1.08	4.9
CFM_IT_2	*Selection system*	0.97	0.97	4.9
PFM_IT	*Retention*	1.20	0.60	4.9
SFM_IT	*Set Aside*	0.00	NA*	4.9
CFM_IE	*Selection system*	0.86	1.47	0.6
PFM_IE	*Clear cut*	1.20	0.60	0.6

[1] This value can be higher than 1 because it is relative to the current management. So if the non-coniferous share increases by 50%, then the share compared to the current value is 1.5.

[2] In set-aside management, no assumptions regarding the non-conifer share were made in the economic modeling, since these areas are not used for wood supply. Therefore, this share was not modeled and did not change compared to the current one (NA).

SFM_IE	*Set Aside*	0.00	NA*	0.6
CFM_LT	*Selection system*	0.97	1.14	11.4
PFM_LT	*Clear cut*	1.20	0.60	11.4
SFM_LT	*Set Aside*	0.00	NA*	11.4
CFM_SK	*Selection system*	0.93	1.14	11.0
PFM_SK	*Retention*	1.20	0.60	11.0
SFM_SK	*Set Aside*	0.00	NA*	11.0
CFM_SE	*Selection system*	0.92	1.32	22.2
PFM_SE	*Clear cut*	1.20	0.85	22.2
SFM_SE	*Set Aside*	0.00	NA*	22.2
CFM_NL_1	*Selection system*	0.92	1.02	4.1
CFM_NL_2	*Selection system*	0.94	0.91	4.1
PFM_NL	*Clear cut*	1.22	0.60	4.1
SFM_NL	*Set Aside*	0.00	NA*	4.1
CFM_PT	*Selection system*	0.90	1.50	3.3
PFM_PT	*Clear cut*	1.20	0.63	3.3
SFM_PT	*Set Aside*	0.00	NA	3.3

DE, IT, IE, LT, NL, PT, SE, SK = Country of AFM origin (Germany, Italy, Ireland, Lithuania, Netherlands, Portugal, Sweden, Slovakia) where the case studies of the ALTERFOR project were performed. CFM, PFM, SFM= type of Forest Management model (Close-to-nature, Production, Set-Aside).

S3 Definitions of forest management practices

Table S 3.1 *List of forest management practices modeled in the present study*

Clear-cut	Even-aged silviculture practice where the entire stand overstorey is removed in one harvest. The stand is naturally or artificially regenerated afterwards. (temperate and boreal biome).
Retention	Partial harvesting that allows new stems to grow up under an overstory of maturing trees. The shelterwood may be removed at a later date.<br><br>Individuals (dispersed retention) or groups of trees (aggregated retention) are left on-site to maintain structural diversity (such as patch-cut or green tree retention systems), supply seeds for the next crop (seed tree retention) or to protect the regeneration. (temperate and boreal biome).
Selection system	Individual mature trees are harvested (single-tree selection), groups of mature trees (group-selection), or a combination of the two to create small openings scattered throughout the stand. (temperate and boreal biome).
Selective Logging	It involves the removal of the largest, highest quality trees from a tropical forest stand, leaving the remaining vegetation standing. (tropical and subtropical biome).
Reduced Impact Logging (RIL)	Attempt to manage tropical forests for timber in a more sustainable way by reducing the damage to soil, future crop trees, residual stands, and workers (e.g., through detailed harvest plans and worker education, training and supervision). RIL has been conceived to achieve a sustainable level of harvest, but also to improve sustainability in terms of biodiversity, carbon retention and ecosystem services. (tropical and subtropical biome). [1]
Timber plantation	Monocultures of fast-growing species planted and harvested in short time cycles (20-40 years) for production of timber assortments.
Plantation for pulpwood and fuelwood	Monocultures of fast growing, low-density wood species, such as Pinus spp. or Eucalyptus spp., used for fuel, or the pulp and paper industry. Typically harvested on a much shorter time cycle (5-15 years) than timber plantations.

S4 Mapping of regions in the projections of land use

Inside the EU28, the computation was performed for the 246 European administrative units (NUTS2) (Figure S 4.1 and Table S 4.1). Outside the EU28, the results for each scenario were obtained at the resolution of “GLOBIOM Region”, which divides the globe into 29 trading regions and the EU28 (Figure S 4.2 and Table S 4.2). Additionally, for wood production (i.e., forest harvest), the GLOBIOM results were firstly computed outside the EU28 on a grid of 200x200 km (Land Units, LU) and subsequently upscaled at the resolution of GLOBIOM Regions (i.e., trade regions). To match the model used to compute species loss, all results were re-mapped to the resolution of 779 “Ecoregions”, using GIS spatial intersection of NUTS2/GLOBIOM Regions and the Ecoregions. The area shares of NUTS2/GLOBIOM Region per Ecoregion were then multiplied by the land and products (wood harvested and semifinished products, then converted to roundwood equivalents in the modelling) within each NUTS2/GLOBIOM Region. For wood trade volumes from outside the EU28, firstly an allocation to LUs within each GLOBIOM Region was created, based on the share of wood harvested from each LU within the GLOBIOM Region. Afterwards, the allocation from LUs to Ecoregions was obtained, similar to that described above for other land/products.

Mapping of NUTS2 administrative Regions (EU28)

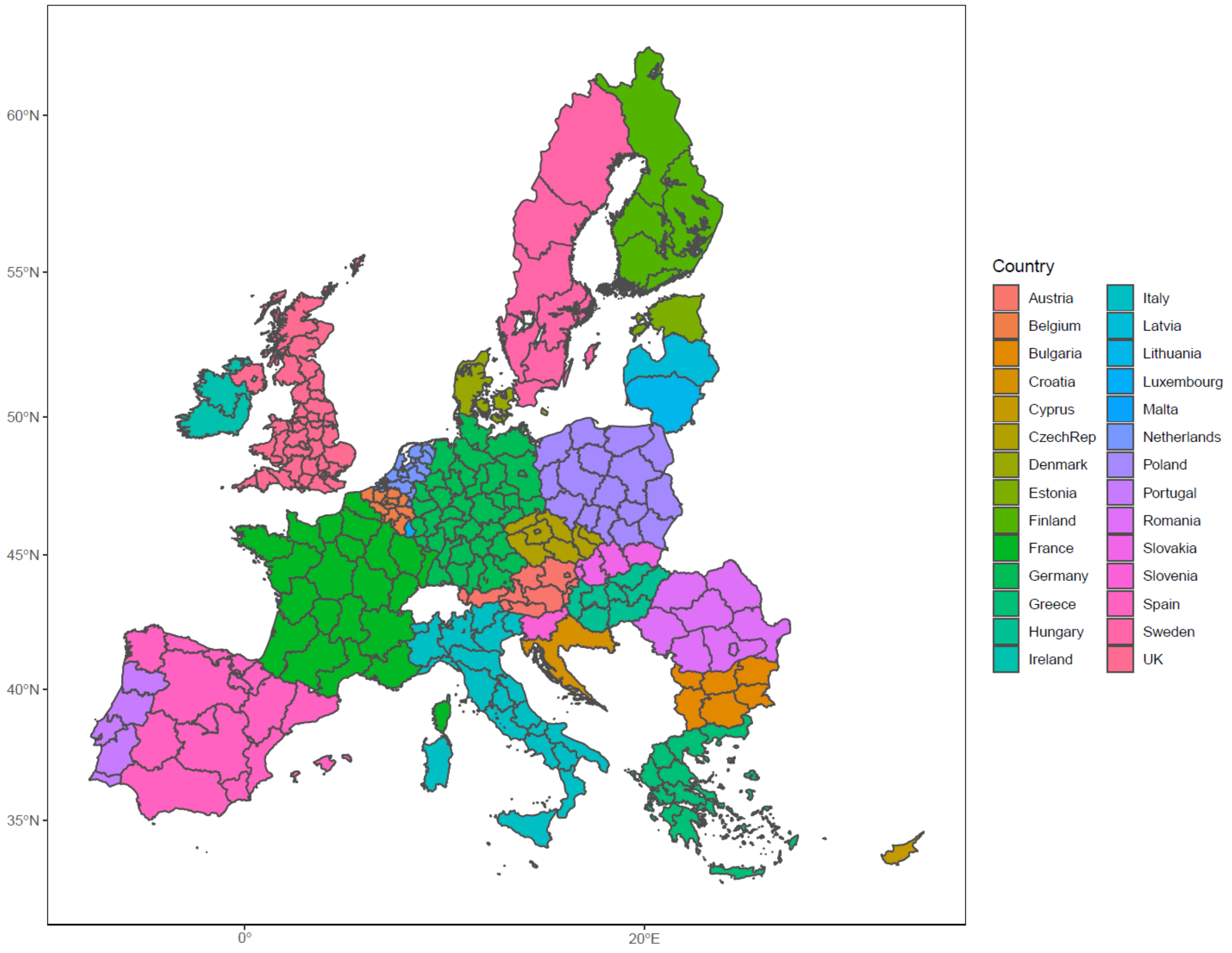

Figure S 4.1 *Map of EU28 NUTS2 regions.*

Table S 4.1 *Matching between NUTS2 and EU28 countries.*

Country	*NUTS2*
Austria	AT11, AT12, AT13, AT21, AT22, AT31, AT32, AT33, AT34
Belgium	BE10, BE21, BE22, BE23, BE24, BE25, BE31, BE32, BE33, BE34, BE35
Bulgaria	BG01, BG02, BG03, BG04, BG05, BG06
Croatia	HR
Cyprus	CY00
Czech Republic	CZ01, CZ02, CZ03, CZ04, CZ05, CZ06, CZ07, CZ08
Denmark	DK00

Estonia	EE00
Finland	FI13, FI14, FI15, FI16, FI17, FI20
France	FR10, FR21, FR22, FR23, FR24, FR25, FR26, FR30, FR41, FR42, FR43, FR51, FR52, FR53, FR61, FR62, FR63, FR71, FR72, FR81, FR82, FR83
Germany	DE11, DE12, DE13, DE14, DE21, DE22, DE23, DE24, DE25, DE26, DE27, DE30, DE40, DE50, DE60, DE71, DE72, DE73, DE80, DE91, DE92, DE93, DE94, DEA1, DEA2, DEA3, DEA4, DEA5, DEB1, DEB2, DEB3, DEC0, DED1, DED2, DED3, DEE1, DEE2, DEE3, DEF0, DEG0
Greece	GR11, GR12, GR13, GR14, GR21, GR22, GR23, GR24, GR25, GR30, GR41, GR42, GR43
Hungary	HU01, HU02, HU03, HU04, HU05, HU06, HU07
Ireland	IE01, IE02
Italy	IT11, IT12, IT13, IT20, IT31, IT32, IT33, IT40, IT51, IT52, IT53, IT60, IT71, IT72, IT80, IT91, IT92, IT93, ITA0, ITB0
Latvia	LV00
Lithuania	LT00
Luxembourg	LU00
Malta	MT00
Netherlands	NL11, NL12, NL13, NL21, NL22, NL23, NL31, NL32, NL33, NL34, NL41, NL42
Poland	PL01, PL02, PL03, PL04, PL05, PL06, PL07, PL08, PL09, PL0A, PL0B, PL0C, PL0D, PL0E, PL0F, PL0G
Portugal	PT11, PT12, PT13, PT14, PT15
Romania	RO01, RO02, RO03, RO04, RO05, RO06, RO07, RO08
Slovakia	SK01, SK02, SK03, SK04,
Slovenia	SI00
Spain	ES11, ES12, ES13, ES21, ES22, ES23, ES24, ES30, ES41, ES42, ES43, ES51, ES52, ES53, ES61, ES62, ES63
Sweden	SE01, SE02, SE04, SE06, SE07, SE08, SE09, SE0A
UK	UKC1, UKC2, UKD1, UKD2, UKD3, UKD4, UKD5, UKE1, UKE2, UKE3, UKE4, UKF1, UKF2, UKF3, UKG1, UKG2, UKG3, UKH1, UKH2, UKH3, UKI1, UKI2, UKJ1, UKJ2, UKJ3, UKJ4, UKK1<br><br>UKK2, UKK3, UKK4, UKL1, UKL2, UKM1, UKM2, UKM3, UKM4, UKN0

Mapping of GLOBIOM Regions

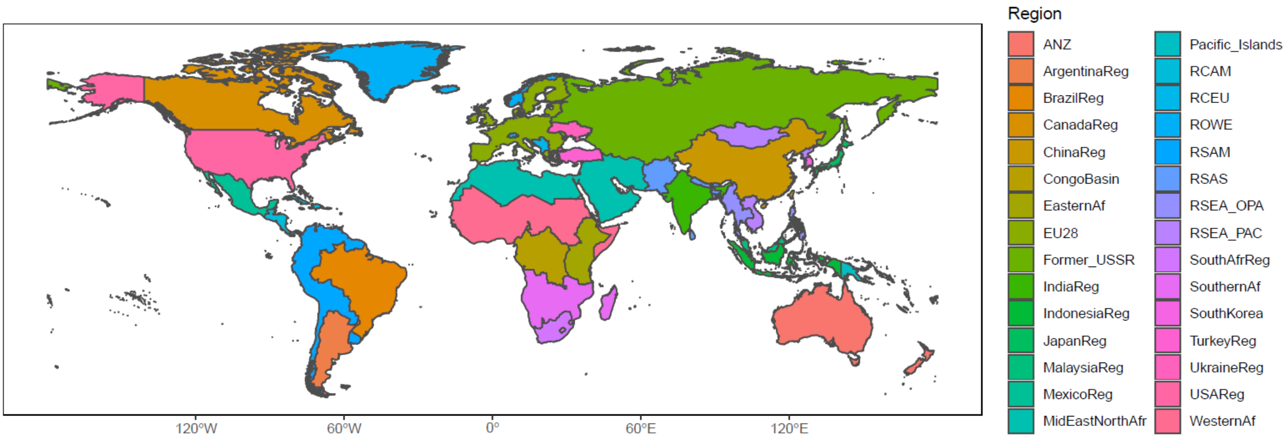

Figure S 4.2 *Map of GLOBIOM regions*

Table S 4.2 *Matching between GLOBIOM regions and world countries.*

GLOBIOM Region	*Country*
ANZ	Australia, New Zealand
ArgentinaReg	Argentina
BrazilReg	Brazil
CanadaReg	Canada
ChinaReg	China
CongoBasin	Cameroon, Central African Republic, Dem Rep Congo, Rep Congo, Eq Guinea, Gabon
EU28	Austria, Belgium, Bulgaria, Cyprus, Czech Republic, Germany, Denmark, Estonia, Spain, Finland, France, Greece, Croatia, Hungary, Ireland, Italy, Lithuania, Luxembourg, Latvia, Malta, Netherlands, Poland, Portugal, Romania, Sweden, Slovenia, Slovakia, United Kingdom
Former USSR	Armenia, Azerbaijan, Belarus, Georgia, Kazakhstan, Kyrgyzstan, Moldova Rep, Russian Fed, Tajikistan, Turkmenistan, Uzbekistan
IndiaReg	India
IndonesiaReg	Indonesia
MalaysiaReg	Malaysia
MexicoReg	Mexico

MidEastNorthAfr	Algeria, Bahrain, Egypt, Iran, Iraq, Israel, Jordan, Kuwait, Lebanon, Libya, Morocco, Oman, Palestine, Qatar, Saudi Arabia, Syria, Tunisia, United Arab Emirates, West Sahara, Yemen
Pacific_Islands	Fiji Islands, Fr. Polynesia, New Caledonia, Papua N. Guinea, Samoa, Solomon Is., Vanuatu
RCAM	Bahamas, Belize, Costa Rica, Cuba, Dominican Rp., El Salvador, Guadeloupe, Guatemala, Haiti, Honduras, Jamaica, Nicaragua, Panama, Trinidad & Tobago
RCEU	Albania, Bosnia Herzegovina, Macedonia, Serbia-Monte
ROWE	Greenland, Iceland, Norway, Switzerland
RSAM	Bolivia, Chile, Colombia, Ecuador, Falkland Is., French Guiana, Guyana, Paraguay, Peru, Suriname, Uruguay, Venezuela
RSAS	Afghanistan, Bangladesh, Bhutan, Nepal, Pakistan, Sri Lanka
RSEA_OPA	Brunei Darussalam, Myanmar, Philippines, Singapore, Thailand, Timor Leste
RSEA_PAC	Cambodia, Korea DPR, Laos, Mongolia, Viet Nam
SouthAfrReg	South Africa
SouthKorea	Korea Rep.
EasternAf	Burundi, Ethiopia, Kenya, Rwanda, Tanzania, Uganda
SouthernAf	Angola, Botswana, Comoros, Lesotho, Madagascar, Malawi, Mauritius, Mozambique, Namibia, Reunion, Swaziland, Zambia, Zimbabwe
WesternAf	Benin, Burkina Faso, Cape Verde, Chad, Cote d'Ivoire, Djibouti, Eritrea, Gambia, Ghana, Guinea, Guinea Bissau, Liberia, Mali, Mauritania, Niger, Nigeria, Senegal, Sierra Leone, Somalia, Sudan, Togo
TurkeyReg	Turkey
UkraineReg	Ukraine
USAReg	USA, Puerto Rico
JapanReg	Japan

S5 GLOBIOM model for the projections of the global land use module

The global model allows for the development of different land uses according to the two climate scenarios, while fulfilling the demands in agriculture and forestry sectors in the global market. This model projects land use change in the following categories: "primary forests", "managed forests", "afforestation", "energy crops", "energy plantations", "cropland", "grassland", "other agricultural land", and "other natural vegetation". Afforested lands are part of the climate mitigation strategy, and they refer to forests originating from land use abandonment (cropland, grassland) or the development of natural vegetation areas. Primary forests and other natural vegetation were considered reference ecosystems in each ecoregion, under the assumption of negligible human impacts. Originally in the model, “grassland” referred only to grasslands under production for feeding animals and did not include “rangelands” (i.e., low-intensity managed pastures), which were under the category “other natural vegetation”. Here, by contrast, we distinguished between “other natural vegetation” and “rangelands”. We set the global area of the latter according to FAOSTAT[2] and merged it with the “grasslands” category. Based on FAOSTAT[2] data, we assumed that rangelands and grasslands remained a constant share (51%) of the other natural vegetation area. Finally, “urban land” was added to the land uses provided by GLOBIOM according to LADA and Anthrome maps[3,4]. There were two additional land categories; “not relevant” land (i.e., relatively depauperate land such as deserts, glaciers), and wetlands; for which development was not modeled and these areas were not included in the calculation of the impacts on species richness. Table S5.1 contains all the land use categories modelled in the study and the corresponding classification in the biodiversity model.

Table S5.1 *Classification of land use categories and forest management practices in GLOBIOM or in other data sources (left column) and the corresponding classification for the response ratios available in the model for the species loss assessment (right column). The symbols in front of the categories distinguish i) between land use types in GLOBIOM global module (•) and EU-module (◊) (left column) and ii) between different data sources († [5,6], ° [1], ▪ [7,8]) (right column). A double symbol (•◊) means that the classification applies both in the global and in the EU-module. The square symbol (□) means that the land use category was not modelled in GLOBIOM but came from another data source.*

GLOBIOM land use categories	Response ratios[1,5,6]
• Cropland	† Annual crops
• Grassland	† Pastures
• Other agricultural land<br>•◊ Lignocellulosic energy crops already used for energy production – internal EU28<br>◊ Lignocellulosic energy crops converted from other land use types – internal EU28	† Permanent crops
◊ Clear-cut – internal EU28 and imported into the EU28 through trade	° Clear-cut
◊ Retention system - internal EU28	° Retention
◊ Plantations for energy wood already used for energy production – imported into the EU28 through trade<br>◊ Timber plantation and plantation for pulpwood – imported into the EU28 through trade<br>◊ Plantations for energy wood converted from other land use types – imported into the EU28 through trade	° Timber plantation and plantation for pulpwood and fuelwood
◊ Selection system – internal EU28	° Selection system (temperate and boreal)
•◊ Low intensity managed forest	° Merging of selective logging, selection system and retention
•◊ High intensity managed forest	° Merging of clear-cut, timber plantations, non-timber plantations and plantations for fuelwood and pulp
• Afforested land<br>•◊ Forest regrowth	▪ Secondary forest<br>The response ratios for this category were obtained by combining the results of a meta-analysis on biodiversity response in secondary forests[7] and the modelling framework applied therein with the model defined in a more recent study on recovery trajectories[8] (see S6 for the details).
• Primary forest<br>• Natural land	Remaining natural land
Not relevant and wetlands	-

From other land use data sources	
□ Rangeland[2]	† Pastures
□ Urban[3,4]	† Urban

S6 Response ratios for secondary forests

To quantify the response ratios for recovered areas, we use the results of a meta-analysis which collected recovery times for forests per species group and ecoregion and estimated some parameters relevant for modelling the process (e.g., the age coefficient which characterizes the relationship between time and response ratio, hereafter *n* [7]). The reference cited assumed a linear recovery and developed a model accordingly. Here, we used the same input data but applied a more accurate modelling framework , which is , based on a logarithmic function that better describes the trajectory of the response ratios over time[8]. We followed the process outlined in Figure S6.1.

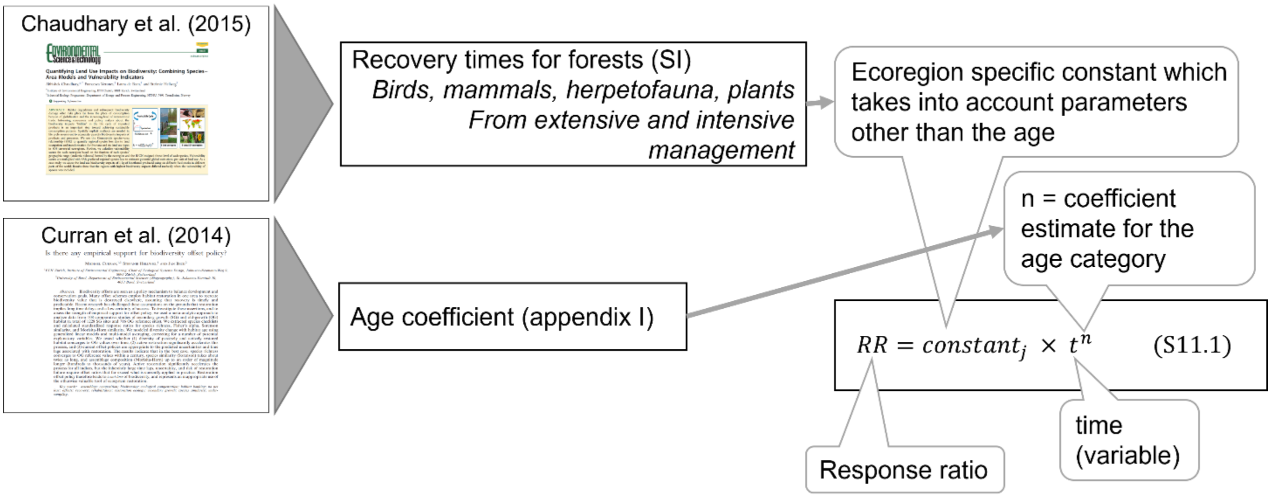

Figure S 6.1 *Outline of the process used to obtain the response ratios for secondary forests (afforested and regrowth lands).*

The equation shown in Fig. S6.1:

$$RR = constant_j \cdot t^n \qquad \text{Eq. S6.1}$$

was used to derive $constant_j$. For each ecoregion, $constant_j$ can be computed considering that RR is equal to 1 when t equals the recovery time ($T_{recovery,\,j}$), therefore $constant_j = 1/(T_{recovery,\,j})^n$. $constant_j$ allowed us to compute the RR when t was not equal to the recovery time. As Curran *et al.* (2014)[7]

found that the coefficient estimate for the age category (*n* in eq. S6.1) is equal to 0.01 (Appendix I and Appendix B of Curran et al. (2014)[7]), it was possible to draw the new trajectory defined by eq. S6.1 (Figure S6.2). The shortest and the longest recovery times[5,7] were taken and applied to the Eq. in Fig. S6.1 (70 and 1200 years respectively). The curves start at time = 1 year after the beginning of the recovery (at 0 years it would be at 0 since data on RR for the and use from which the recovery is done were not available).

The graphs show that the recovery is very fast, and after few years, it is already above 95%. We thus decided to take the black curve as a reference (conservative approach) and calculate the average of it as a response ratio (0.957).

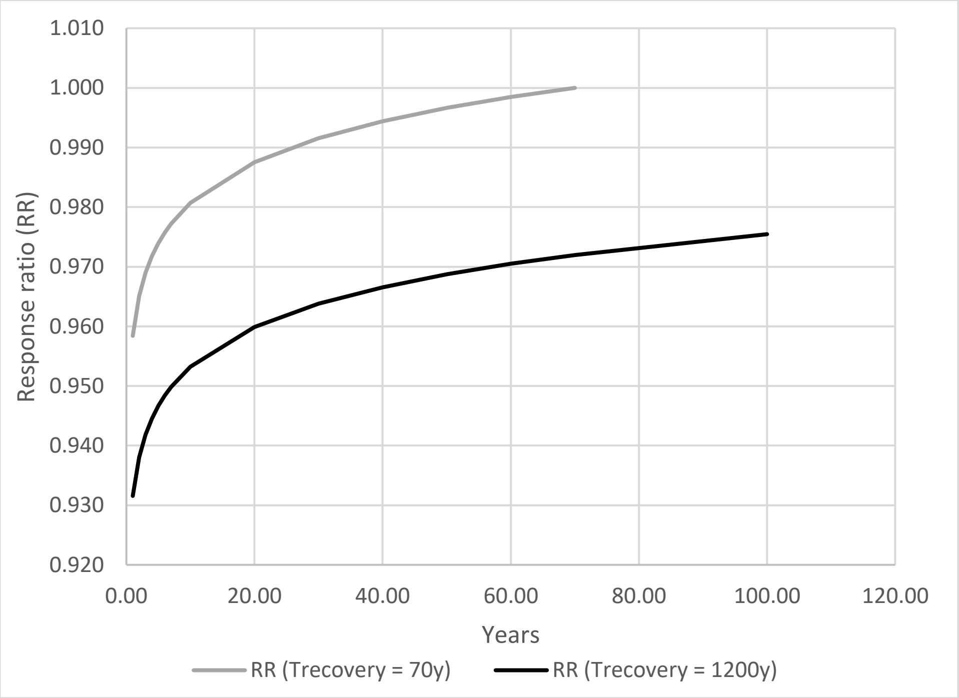

Figure S 6.2 *Trajectories of the response ratios considering the smallest recovery time (light grey curve) and the largest recovery time (black curve).*

S7 Allocation of land use areas

The mapping between the areas in the global module and the EU-demand-module in GLOBIOM was performed using several allocation procedures. In the global module, this operation concerned the category “managed forest”, “annual crops”, “grasslands”, “natural lands”.The global module defined the development of these land uses at the level of GLOBIOM Region and the EU-module was used for downscaling the allocation of different land use areas to the Ecoregions.

The areas occupied by forest use intensities defined in the EU-demand-module were subtracted from the category “managed forest” in the general model. On the one hand, as the module providing the forest management intensities included only the areas used to meet the EU28 forest biomass demand, some ecoregions had remaining areas of “managed forests”. On the other hand, due to the mapping of GLOBIOM regions to ecoregions, the matching was not accurate in all the ecoregions and, in a few ecoregions, the manged forest areas from the EU-demand-module were larger than the available forestry area in the global-module . These values were then allocated to the remaining surplus areas of “managed forest” within the same GLOBIOM region (Figure S7.1). Afterwards, ifn those ecoregion where there was still a surplus of “managed forest” areas in the global module, an allocation to three broad forest use categories “intensively managed forests”, “low-intensity managed forests” and “regrowth” was performed according to FAO data[2]. To do this, the corresponding raw response ratios were merged according to the aggregation described in Table 2.

The same issue came up when the energy crops/plantations of the EU-demand-module were sub-tracted from the corresponding land type that were converted (annual crops, grasslands, natural lands). Similarly, the exceed area of the EU-demand-module was allocated to the remaining surplus areas of the land use types involved in the conversion within the same ecoregion (if enough area was

available), or within the same GLOBIOM region (global module), according to the shares of the converted areas from each land use type (Figure S 7.2).

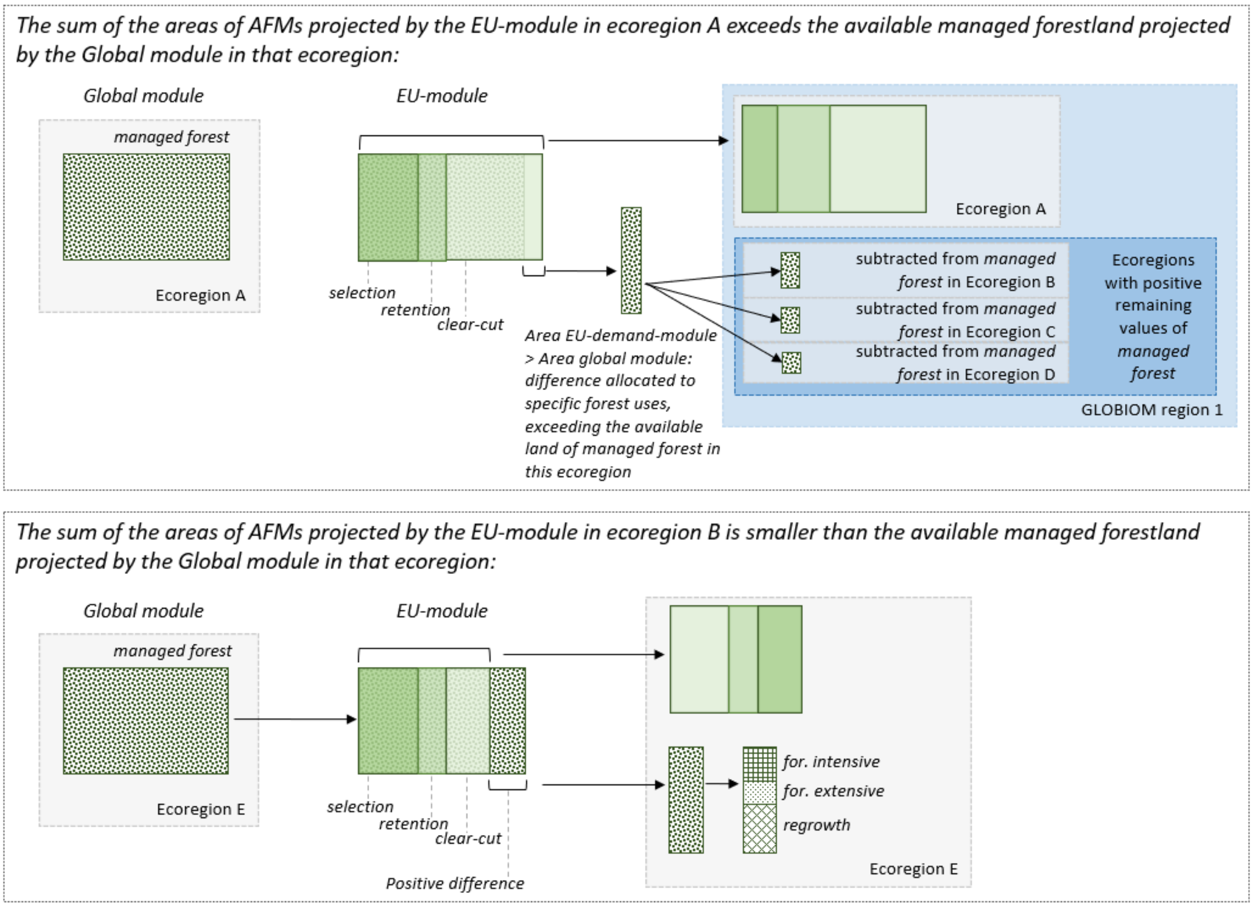

Figure S 7.1 *Allocation of managed forests in the matching between the global module and the EU-module*

Example. The land projected by the EU-module to be converted from natural land to lignocellulosic crops or energy plantations in ecoregion A is larger than the area projected by the global module to be natural land in ecoregion A.

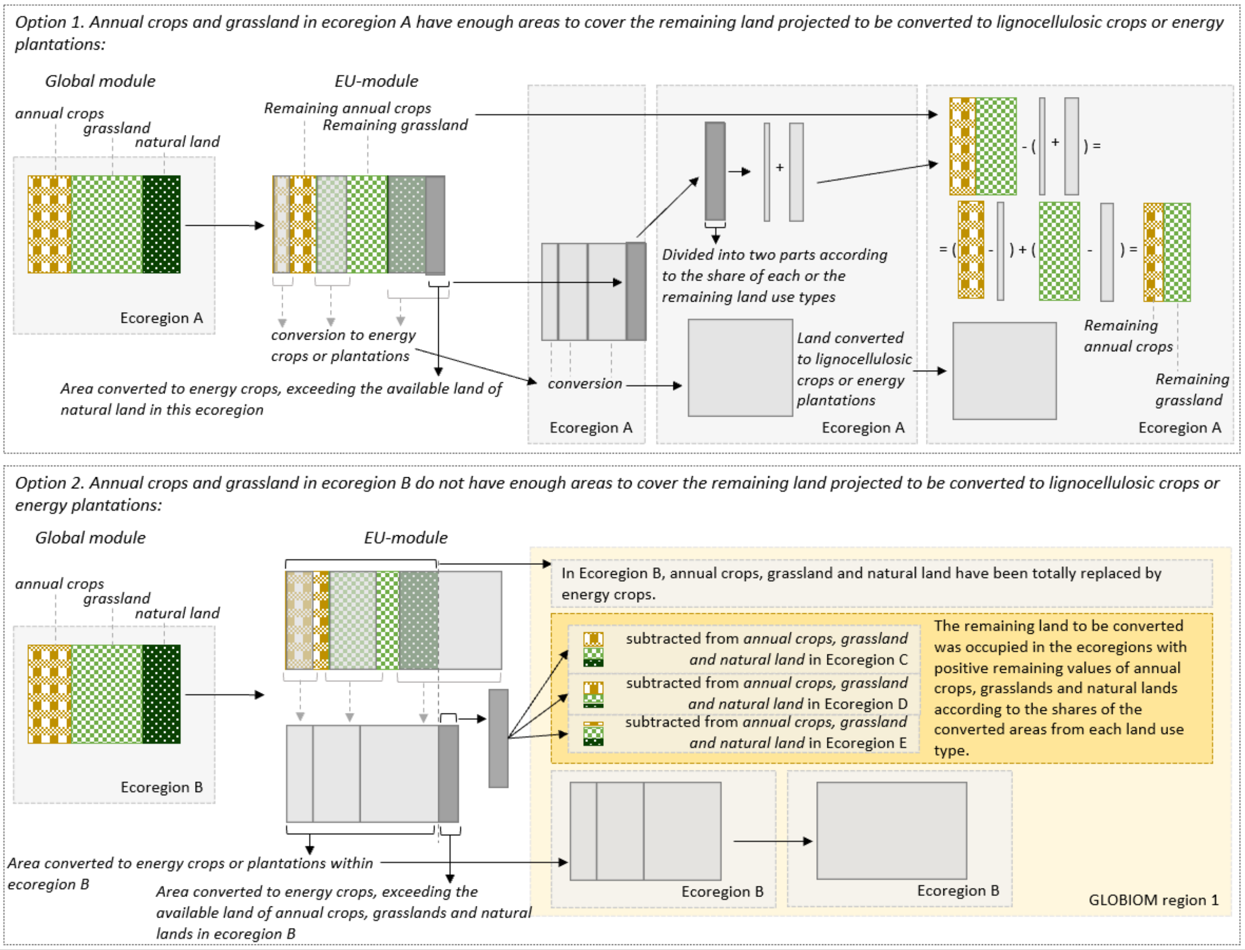

Figure S 7.2 *Allocation of annual crops, grasslands and natural lands when matching between the global module and the EU-module for energy crops and plantations.*

S8 Modelling of species loss – methodological detail

Species loss due to human land use is evaluated using the countryside species-area relationship (countryside-SARs)[9], which recognises that some species will survive even in human-transformed landscapes, according to the species habitat requirements[10,11]. Estimating the potential disappeared fraction of species involved several steps[5,6]. First, 1a) species loss was quantified at regional level per species group and 1b) allocated to each land use type. Then, 2) the loss at ecoregion level was multiplied by the vulnerability scores of each species group and ecoregion to obtain global species loss and being able to compare losses occurring in different regions. These scores considered the endemicity, range and threat level of all species occurring in an ecoregion. Finally, 3) the results were converted to global potentially disappeared fraction of species (PDF) by dividing them by the global number of species belonging to each species group. The last step consisted in aggregating the global PDF across multiple species groups. The detailed description of each step (which was based on the methodology recommended by UNEP-SETAC Life Cycle Initiative[5,6,12]) is presented here below.

1a) At the regional level, species loss was assessed according to the following equation:

$$S_{lost,regional,t,j,} = S_{org,t,j}\left[1-\left(\frac{A_{new,j}+\sum_{i=1}^{n} h_{t,i,j}A_{i,j}}{A_{org,j}}\right)^{z_j}\right] \qquad \text{(S8.1)}$$

Parameter	Description
$S_{org,t,j}$	Number of species of species group t in undisturbed natural habitat in ecoregion j[5].
$S_{lost,regional,t,i,j}$	Number of species lost in ecoregion j for species group t. It corresponds to the difference between $S_{org,t,j}$ and the number of species occurring in the human-modified land use (calculated).
$A_{new,j}$	Remaining natural habitat area in ecoregion j from GLOBIOM[13–15].

$A_{i,j}$	Area occupied by land use type i in ecoregion j from GLOBIOM[13–15] (except the Urban land use type[3,4]).
$A_{org,j}$	Original natural habitat area from GLOBIOM[13–15].
$h_{t,i,j}$	Affinity of species group t to land use i in ecoregion j (for annual crops, permanent crops, pastures and urban[5]; for management intensities[1]).
z_j	Parameter of SAR for ecoregion j[16].

The term $h_{t,i,j} \times A_{i,j}$ allowed us to also consider the contribution to species richness from those land use types that are not natural, and this is what distinguishes the countryside SAR from the classic SAR. Additionally, as pointed out in the previous section, applying the model directly to GLOBIOM areas maintained the non-linear relationship between area extent and species lost.

To obtain the factor h, the response ratio (RR) was needed. The response ratio is the ratio between the number of species occurring under a specific land use type and the number of species in the reference state (unmanaged forest sites) for different species groups. The RRs were consequently used to compute the affinity of species groups to land use types.

$$h_{t,i,j} = \left(RR_{local,t,i,j}\right)^{\frac{1}{z_j}} \qquad \text{(S8.2)}$$

where RR is the response ratio per species group *t*, land use type *i* and ecoregion *j*[1,5].

Equation S7.2 was derived as follows.

According to the definition of the countryside Species-Area relationship as in Pereira et al. 2014[9], the richness of species group *t* in a certain region is given by

$$S_t = c_t\left(\sum_{i=1}^{m} h_{t,i}A_i\right)^{z_t} \qquad \text{(S8.3)}$$

where $h_{t,i}$ is the affinity of species group *t* to habitat *i*, A_i is the area of habitat *i* in the landscape and *m* is the number of habitat types, and c_t and z_t are the usual parameters of the classic SAR.

If a single habitat type is assumed

$$S_{t,i} = c_t\left(h_{t,i}A_i\right)^{z_t} \quad \text{(S8.4)}$$

We can reverse the equation to derive $h_{t,i}$:$h_{t,i} = \frac{S_{t,i}^{\frac{1}{z_t}}}{c_t^{\frac{1}{z_t}}A_i}$

(S8.5)

The denominator of the equation above corresponds to $S_{t,i,org}^{\frac{1}{z_t}}$, according to the definition of the classic SAR:

$$h_{t,i} = \left(\frac{S_{t,i}}{S_{t,i,org}}\right)^{\frac{1}{z_t}} \quad \text{(S8.6)}$$

$S_{t,i,j}/S_{org,t,j}$ is the response ratio RR for habitat i and species group t. In a natural habitat, the numerator and the denominator are equal and $h_{t,i}$ is 1. Otherwise, if the area is a human-modified landscape, h will be smaller than 1 (but still above 0).

The RR per ecoregion, species group and land use type was calculated as median of the raw values provided in the reference; the detailed description and assumptions made to obtain them are available in sec. S9.

1b) The allocation of regional damage to different land/forest use types was done by multiplying the species lost by an allocation factor a. This factor takes into account the share of each land use per ecoregion and the affinity of each species group to it:

$$S_{lost,regional,t,i,j} = S_{lost,regional,t,j} \times a_{t,i,j} \quad \text{(S8.7)}$$

where $a_{t,i,j}$ is specific of species group t, land use type i and ecoregion j and equal to:

$$a_{t,i,j} = \frac{A_{i,j}(1-h_{t.i.j})}{\sum_{i=1}^{n} A_{i,j}(1-h_{t,i,j})} \quad \text{(S8.8)}$$

The formula above weights the allocation such that land use types with higher impacts have a higher share of species loss for the same area share.

2) $S_{lost,regional,t,i,j}$ does not consider the extent of species range or their conservation status in each ecoregion. In other words, it does not distinguish between common species and threatened species,

nor does it account for global extinction risks, which is important for global assessments.

Regional species loss was therefore weighted with vulnerability scores per species group (t) and ecoregion (j):

$$S_{lost,global,t,i,j} = S_{lost,regional,t,i,j} \times VS_{t,j} \quad \text{(S8.9)}$$

$VS_{t,j}$ depends on species endemicity and threat level (TL, which range trom 0.2 – least concern – to 1 – critically endangered)[17]. Due to a lack of data, the original methodology[6] assumed a TL equal 1 for plants, which sets all plant species to a critically endangered status. Nevertheless, a following study[18] assumed TL equal to 0.5, since plants are known to have a threat status similar to mammals', whose world average is 0.47. We therefore also applied a TL of 0.5 for plants in this study.

The unit of $S_{lost,global,t,i,j}$ is the number of global species equivalent (eq.) lost and is calculated for each species group separately.

3a) To convert global species eq. lost to global PDF, conversion factors are needed. The conversion factors were defined by Eq. (S8.10) and they considered world average vulnerability scores and total global species richness for each species group.

$$C_t = \frac{1}{S_{t,world} \times VS_{t,world}} \quad \text{(S8.10)}$$

In the denominator, $S_{t,world}$ was needed to obtain the fraction of species, whereas $VS_{t,world}$ is included to make sure that the maximum fraction lost is 100%. For mammals, $VS_{mammals,world}$ is 0.44 and $S_{mammals,world}$ is 50'490; for birds $VS_{birds,world}$ is 0.29 and $S_{birds,world}$ is 10'104; for plants $VS_{plants,world}$ is 0.5 (which was adapted to the new threat level value of 0.5) and $S_{plants,world}$ is 321'212.

3b) Finally, the aggregation of the different species groups is performed (S8.11), using a weighting factor (0.33). This factor gives the same weight to all species groups.

$$S_{lost,global,aggr.,i,j} = 0.33 \cdot \left(\sum_{t=1}^{N} S_{lost,global,t,i,j} \times C_t\right) \quad \text{(S8.11)}$$

$S_{lost,global,aggr.,i,j}$ is expressed as PDF and provides a single indicator per ecoregion and land use type,

which estimates the fraction of global species that is projected to go extinct.

The countrysideSAR was applied to 779 out of the initial 804 ecoregions, according to GLOBIOM mapping, which only included potentially productive regions and excluded polar zones, lakes, and the Himalaya (Figure S11.1).

S9 Raw data used in the calculation of the response ratios and the z values

This section describes how the raw data were prepared and aggregated to be used in the model.

The values of response ratios per ecoregion j, species group t and land use i ($RR_{local,t,i,j}$) to be used as input parameters in the model were obtained from raw data coming from two existing studies[1,5]. In both studies, data points useful to derive RRs at different resolutions from existing datasets, meta-analysis or field studies were collected and made available.The calculations of RRs for the broad land use types (annual crops, permanent crops, pastures, urban) and for forest management intensities differed slightly. The former were available per species group, land use type and biome, and were expressed as local characterization factors (relative decrease of species richness)[5] (the RRs can be found in the SI02 of the reference, excel sheet *CF_local*, column *Local_CF*). Since, by definition, CF_{local} is 1 – RR, response ratios were derived as 1 - CF_{local}. For each combination of species groups, land use and biome, RRs were calculated as median of the raw values. If less than five data points were available, an additional level of aggregation was performed, first aggregating data over biomes (therefore having only combinations of land use types and species groups). If data points were still less than five, raw data were aggregated over species groups as well. Since each ecoregion belongs to a biome, data were downscaled accordingly. In addition, for this study, no biome was assigned to urban land use type. The data source we used[5] did not provide information on biomes for artificial lands for 164 out of 190 data points, and the remaining data points cover only plants and few biomes; therefore, to avoid inconsistency, all the data were grouped under the same category.

Regarding forest management intensities, data are provided per species group at continental resolution[1] (the raw data can be found in the SI of the reference, excel sheet *Raw data*, columns *Xc* and *Xe*). Response ratios were obtained as the ratio between values in Xe (“mean species richness in disturbed (managed) forest sites”) and values in Xc (“mean species richness in reference (unmanaged) forest

sites"). Nevertheless, values were aggregated at global level, because for many combinations of land use types and species groups, at the continental scale too few data are available. Similarly, to what was done for the broad land use types, if less than 5 data points were available per forest use intensity and species group, an aggregation was performed. In this case, however, raw data were aggregated and allocated either to low-intensity or high-intensity forest management as described in Table 2 and in Table S.1 of the main text. If the concerned forest use belonged to the plantation category (Timber plantation or Plantation for fuelwood), the aggregation was done over all plantation categories.

The countryside SAR is not meant to account for positive effects on species richness (i.e., an increase of species richness), as explained for the matrixSAR[19](an approach similar to the countryside SAR). Therefore, to prevent the response ratios from being negative, a cutoff was introduced. Before aggregation, all RRs above 1 were set to 1 (as also done in previous studies[1]) and no AFM resulted in a negative PDF, as a RR higher than 1 would mean that more species than those in the natural habitat are accounted for, and whether this is a benefit is still debated. Regarding the z values, we adopted a slightly different approach compared to the previous studies[5,6,19,20]. In previous studies, the average values and the 95% confidence intervals in figure 1 of Drakare et al. (2006)[16] were used (in most cases, the CI were used as minimum and maximum of a triangular distribution to propagate the uncertainties). For this study instead, even though the source of data was the same, we accessed the complete set of raw z values which resulted from the meta-analysis conducted by Drakare et al. (2006)[16] and then excluded the data of water-based habitat types and of the species groups not modelled in the present study. As previously done, we grouped the selected data according to the habitat type (forest, non-forest, islands, as in Figure S 9.1). Ideally, we would have performed an additional sub-classification and also grouped the data according to the species group, however, for mammals there were not enough data for the non-forest ecosystem, so we decided to keep them together. The availability of raw data allowed us to have a base from which to perform the uncertainty analysis (as

described in Sec. S10), and to select only those values which referred to the species groups we are taking into account (whereas previously the values were averaged over all species groups, which included many species groups not considered in this study). The raw data for the z values which were used in Drakare et al. (2006)[16] were kindly sent to us by the corresponding author of the meta-analysis as personal communication via email on May 5th, 2021.

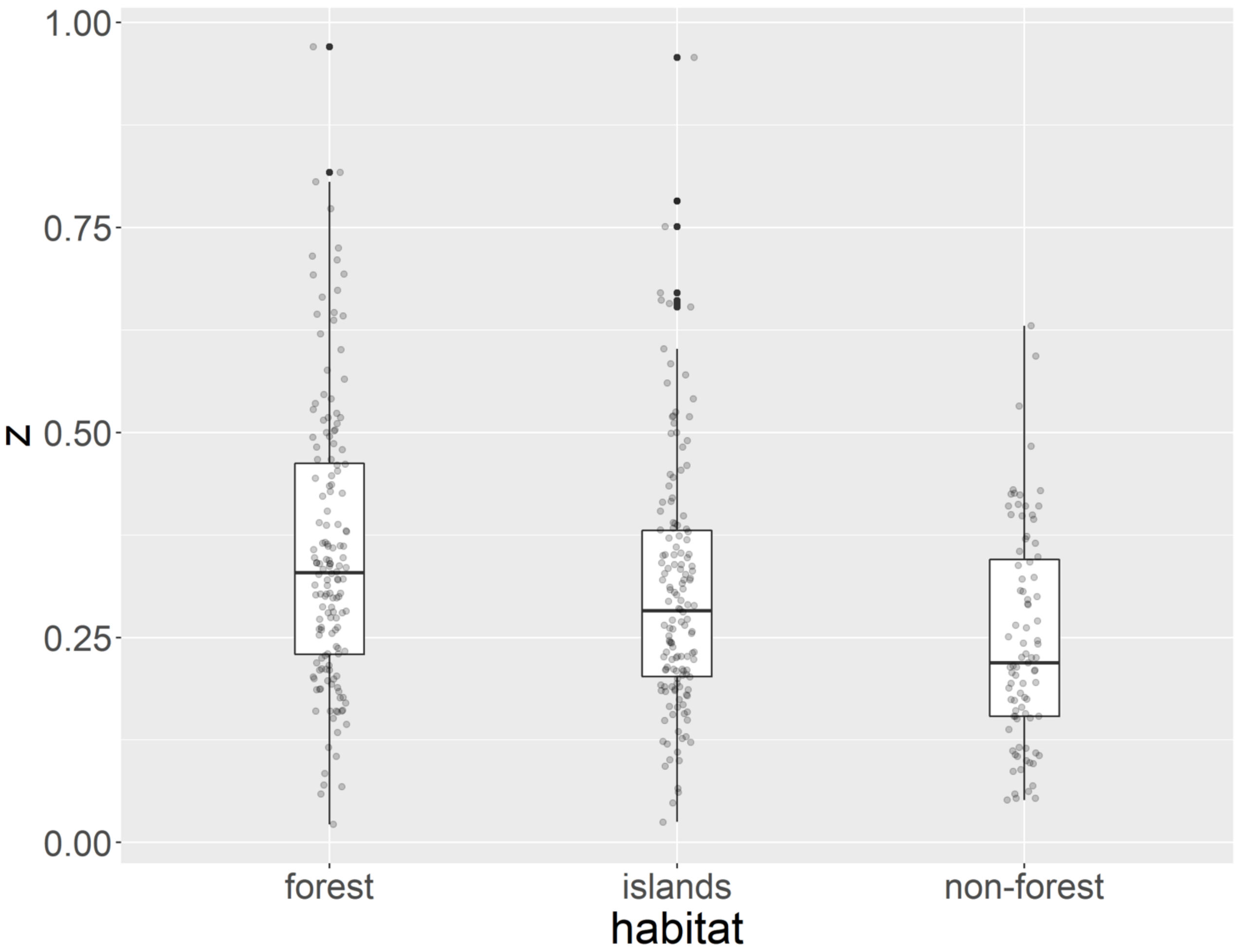

Figure S 9.1 *Boxplots with the z values grouped by habitat type*[16].

S10 Bootstrapping and propagation of uncertainties

Once the raw data were prepared and aggregated to be used in the model as described in Sec. S8, we applied bootstrapping to estimate the confidence intervals of the results. Here below we provide a description of the process, which is also depicted in Figure S 10.1.

Bootstrapping was applied to the raw values of the response ratios (grouped per species group, land use type and ecoregion, according to the assumptions described in Sec. S7) and the raw z values. For both of these parameters, 10,000 median values were sampled using the R function *boot* from the homonymous package. Subsequently, the 10'000 values of RR and z were randomly paired to calculate 10'000 values of $S_{lost,global,aggr.,i,j}$ (eq. S8.11), which corresponded to the bootstrapped distribution of $S_{lost,global,aggr.,i,j}$. As a last step, the confidence intervals were obtained as

$$\left[2\hat{S}_{lost,\,global,\,aggr.,i,j} - q_{0.975}\,,\, 2\hat{S}_{lost,\,global,\,aggr.,i,j} - q_{0.025}\right]$$

where $\hat{S}_{lost,\,global,\,aggr.,i,j}$ is the estimate of $S_{lost,global,aggr.,i,j}$ based on all real data (not bootstrapped), $q_{0.975}$ and $q_{0.025}$ are the 97.5% and 2.5% quantiles, respectively, of the bootstrapped distribution of $S_{lost,global,aggr.,i,j}$.

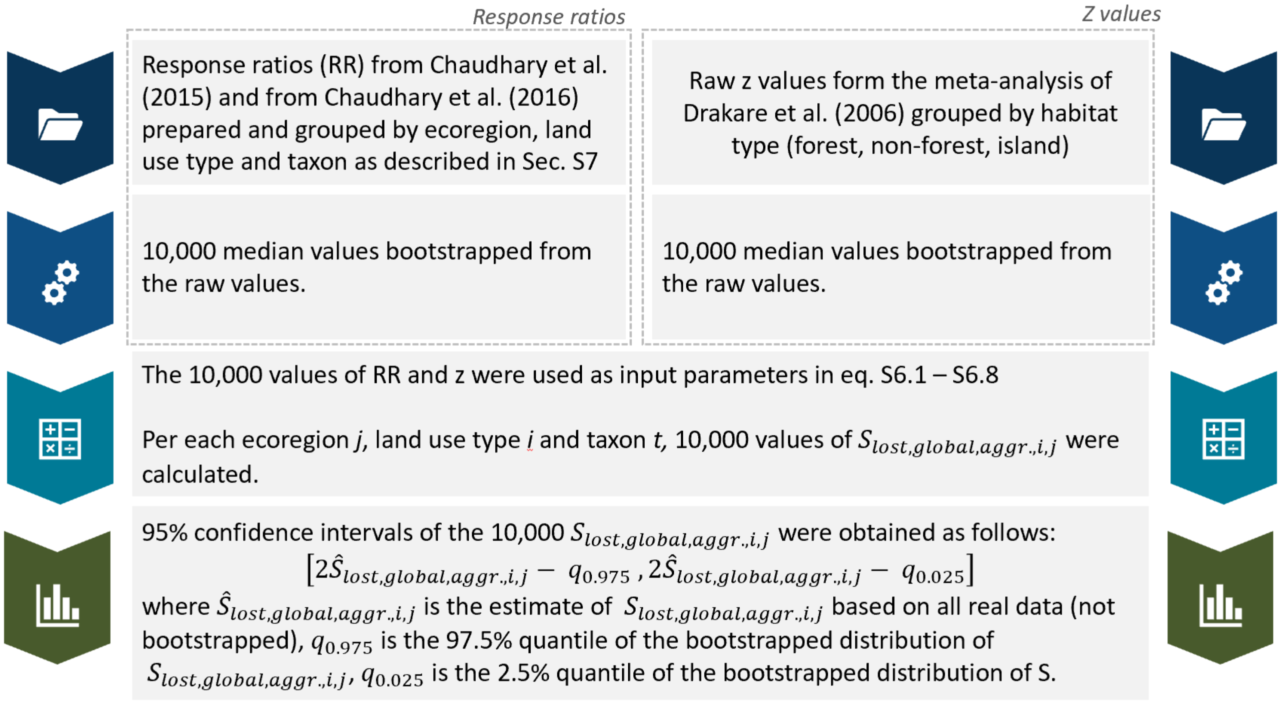

Figure S 10.1 *Bootstrapping process and calculation of* $S_{lost,global,aggr.,i,j}$.

S11 Map of ecoregions included and excluded from the study

The light blue ecoregions in the map here below are not included in the study (this mainly applies to polar zones and lakes (and Himalaya)):

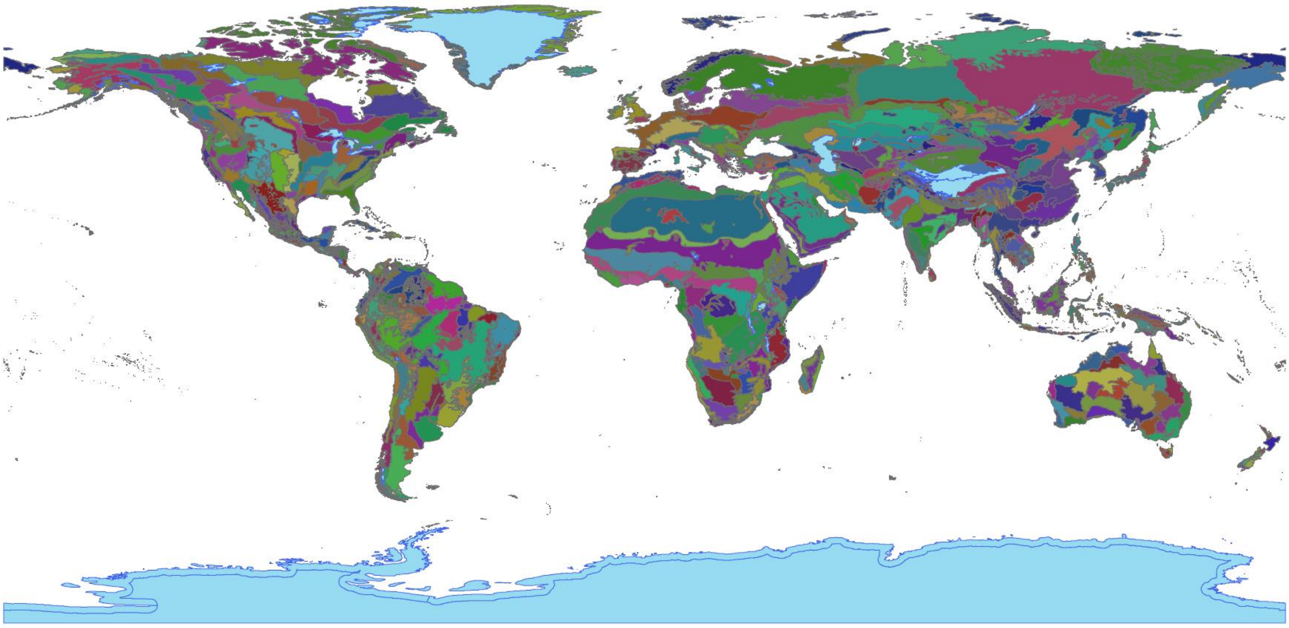

Figure S 11.1 *Global map of ecoregions.*

S12 Sensitivity Analysis

Materials and methods

A sensitivity analysis was conducted to investigate two aspects under the Baseline scenario: how species loss responds to ii) the allocation of part of clear-cut areas in the EU28 to timber plantations, and iii) an extreme forest management scenario (hereafter *laissez-faire*) where the GLOBIOM model was left free to choose the most economically convenient options for the EU28 internal forest management, with very few constraints.

Concerning timber plantations, in the GLOBIOM model part of the areas in the EU28 classified as clear-cut was described as managed with practices potentially more intensive than standard clear-cut practices (plantations of monospecific non-native species). These practices are not as intensive as in the conventional tropical timber plantations, which have a high frequency of harvesting and include the application of pesticides and fertilizers. Nevertheless, it was important to analyse how results could change if these areas were effectively managed more intensively than clear-cut; therefore, part of clear-cut areas was replaced by timber plantations according to the description in the GLOBIOM model. All scenarios were involved in the analysis, exept noCFM/noSFM and CFM50/SFM50, as for these two scenarios all clear-cut areas were described as standard clear-cut.

The last section of the sensitivity analysis examined the effect of the *laissez-faire* scenario on species loss. In this extreme EU28 forest management scenario, current forest management options and AFMs were selected according to economic criteria. This implied that the most productive EU28 forestlands were allocated to intensive management and the share of AFMs was almost zero (0.7 Mha as CFM and 53 Mha as intensive production under RCP6.5, 0.5 Mha as MFM and 65 Mha as as intensive production under RCP2.6).

Results

The following paragraphs analyse the results for 2100.

With regard to the EU28 internal impacts, the replacement of a fraction of clear-cut areas with timber plantations increased the impacts of the default settings of maximum 12% (under RCP6.5 SFM12.5), Figure S13.1.

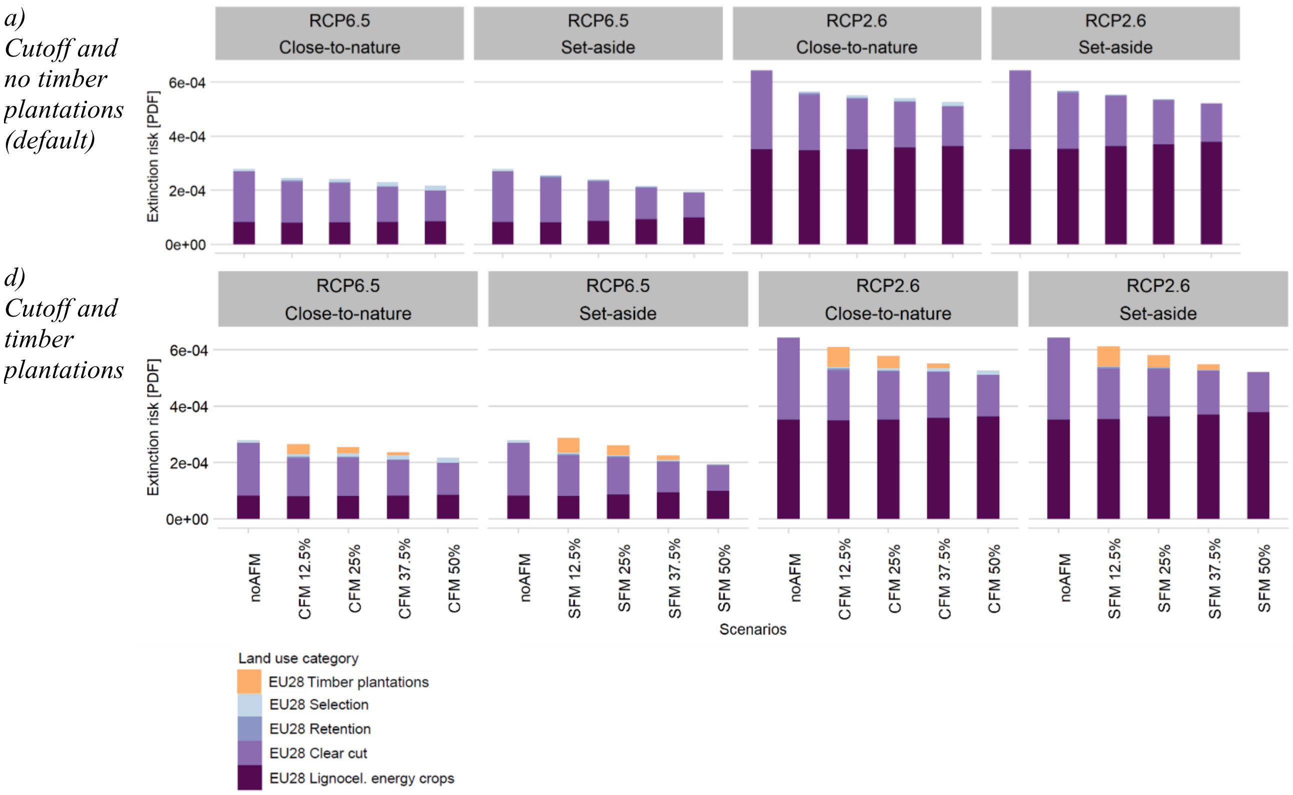

Figure S 12.1 *Sensitivity analysis of the Baseline scenario – Global species extinction risk in 2100 due to EU28 internal forest management and lignocellulosic energy crops for domestic use under different climate and forest management scenarios. The stacked bars display the contributions of the different forest management practices. no AFM = no alternative forest management, CFM or SFM 12.5% / 25% / 37.5% / 50% = close-to-nature management or set-aside implemented on 12.5% / 25% / 37.5% / 50% of EU28 currently managed forestland. Lignocel. = lignocellulosic.*

Considering the impacts at global level, the inclusion of timber plantation caused only a negligible difference.

The results for the extreme EU28 forest management scenario (*laissez-faire*) are described here below.

At the EU28 level, the higher production efficiency compared to noAFM resulted in a smaller area domestically allocated to forestry while achieving the same production outcomes (-3.7 Mha). This led, on average, to a reduced species extinction risk of 12% compared to noAFM, but similar or higher species extinction risk than the CFM and SFM scenarios (up to +27% in RCP6.5 SFM50), as shown in Figure S13.3.

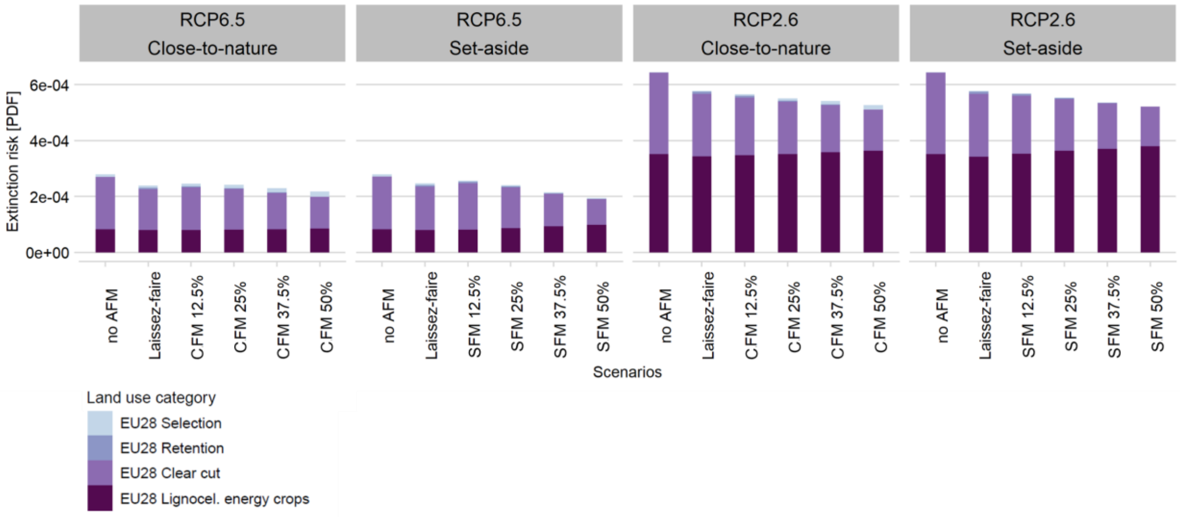

Figure S 12.2 *Sensitivity analysis of the Baseline scenario – Species extinction risk in 2100 due to EU28 internal forest management and lignocellulosic energy crops used for domestic use under different climate and forest management scenarios. The stacked bars display the contributions of the different forest management practices no AFM = no alternative forest management, CFM or SFM 12.5% / 25% / 37.5% / 50% = close-to-nature management or set-aside implemented on 12.5% / 25% / 37.5% / 50% of EU28 currently managed forestland. Lignocel. = lignocellulosic.*

In terms of the total biodiversity footprint, in the projection of species loss for 2100, the laissez-faire scenario showed the lowest values in all cases (Figure S13.4). Under this scenario, the model projected the future EU28 land use according to the most economically advantageous options and therefore selected the most intensive productions. This translated into high EU28 internal wood production. As a result, less wood needed to be imported from forests, plantations, and energy plantations than the other scenarios. The reduction of land used to produce wood imported into the

EU28 from other regions was 24-25 Mha (-38% to -47% for RCP6.5 and RCP2.6, respectively), which translated into a 19 to 28% reduction in species loss compared to noAFM scenario.

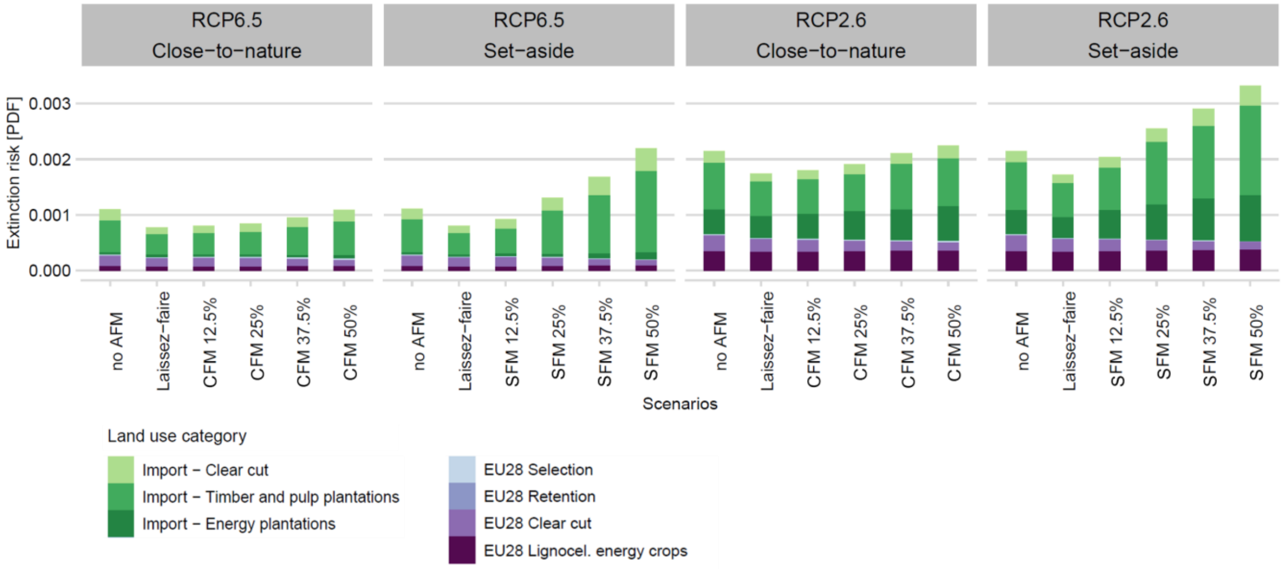

Figure S 12.4 *Sensitivity analysis of the Baseline – Global extinction risk in 2100 due to EU28 demand of forest biomass and lignocellulosic energy crops under different climate and forest management scenarios as result of the sensitivity analysis. The stacked bars display the contributions of lignocellulosic energy crops and managed forests and distinguish between EU28 internal land use for domestic use and imports. no AFM = no alternative forest management, CFM or SFM 12.5% / 25% / 37.5% / 50% = close-to-nature management or set-aside implemented on 12.5% / 25% / 37.5% / 50% of EU28 currently managed forestland. Lignocel. = lignocellulosic.*

S13 Biomass harvested and areas

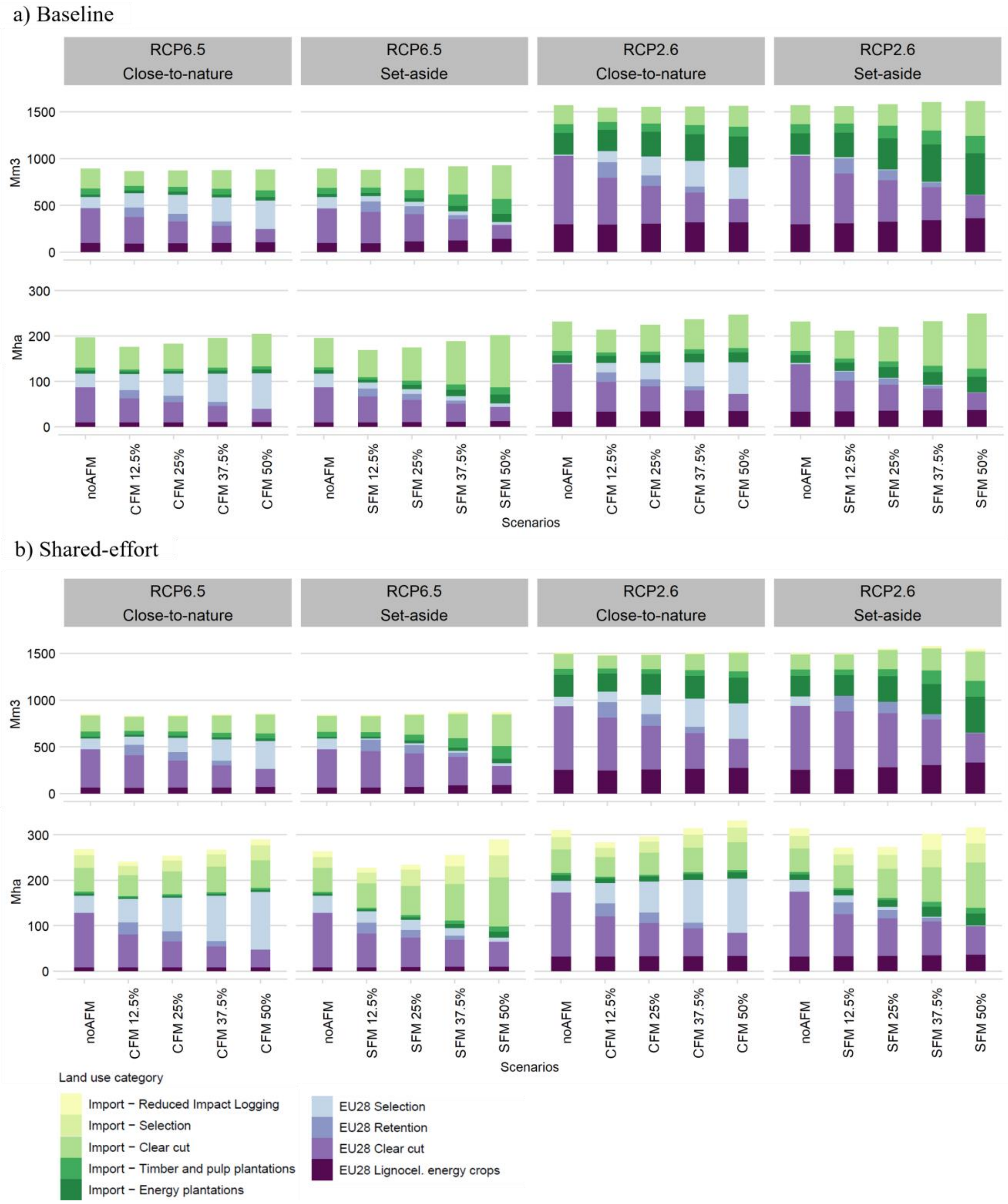

Figure S 13 *Biomass harvested in 2100 for each scenario and for each forest management practice (expressed as Mm3 of roundwood equivalent) and corresponding harvesting areas (in Mha). a) Baseline scenario (internal EU28 managed forestland of 134 Mha and all imports from high-intesity forestry). b) Shared-effort scenario (internal EU28 managed forestland of 160 Mha and part of the imports from low-intesity forestry).*

S14 Development over time of the species extinction risk

EU28 internal forest management

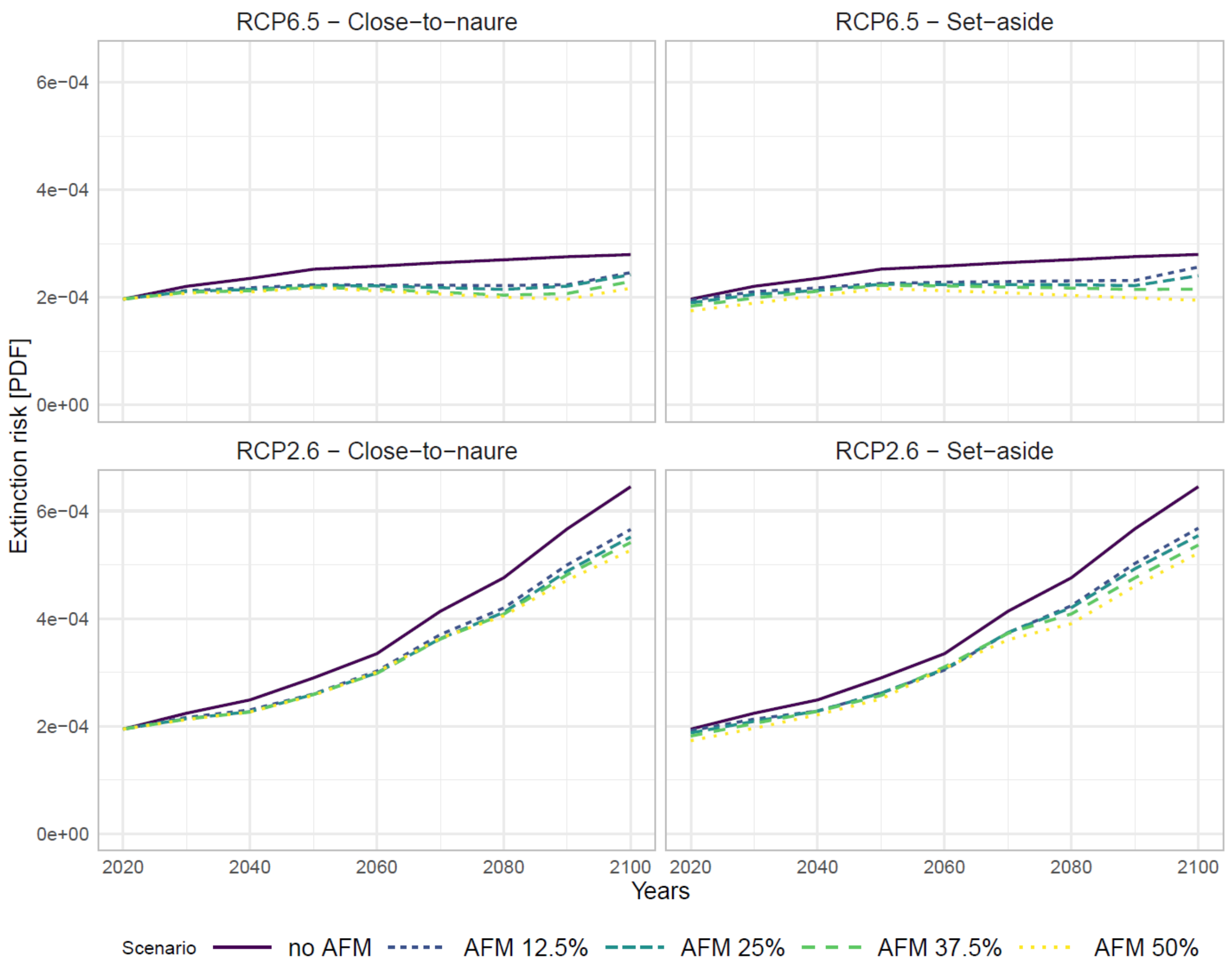

Figure S 14.1 *Baseline scenario – Species extinction risk due to EU28 internal forest management and lignocellulosic energy crops over time under different climate and forest management scenarios. no AFM = no alternative forest management, AFM 12.5% / 25% / 37.5% / 50% = alternative forest management implemented on 12.5% / 25% / 37.5% / 50% of EU28 currently managed forestland.*

EU28 forest biomass footprint

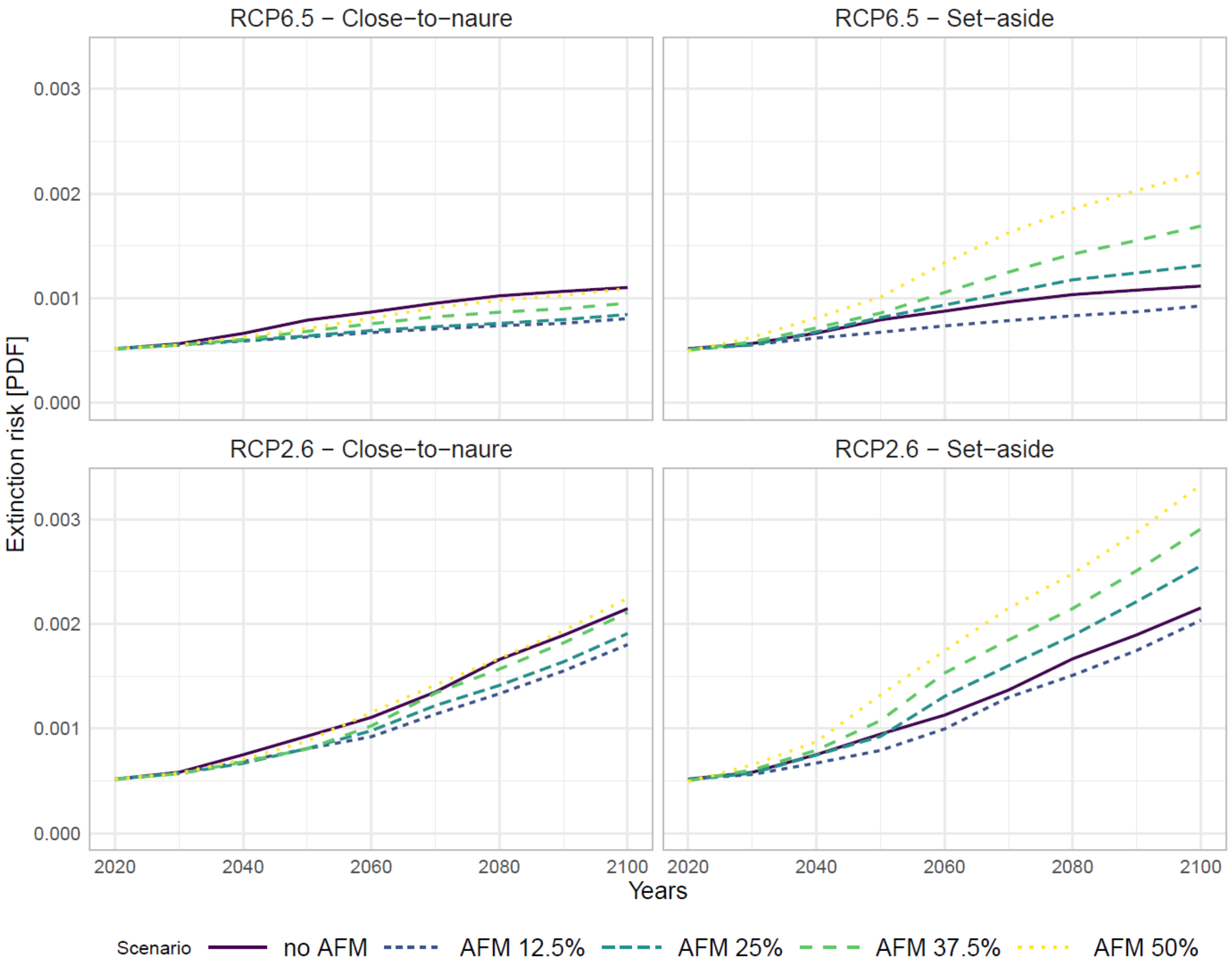

Figure S 14.2 *Baseline scenario - Species extinction risk due to EU28 demand for forest biomass and lignocellulosic energy crops over time under different climate and forest management scenarios. no AFM = no alternative forest management, AFM 12.5% / 25% / 37.5% / 50% = alternative forest management implemented on 12.5% / 25% / 37.5% / 50% of EU28 currently managed forestland.*

S15 Impacts per unit of imported volume

Table S 15.1 *Extinciton risk per millions of cubic meters (PDF * year /Mm3) of wood harvested outside the EU28 in 2100.*

Climate scenario	*Forest use scenario*	*Management scenario*	*Baseline Imported forest biomass (excluding energywood)*	*Baseline Imported energywood from plantations*	*Shared-effort Imported forest biomass (excluding energywood)*	*Shared-effort Imported energywood from plantations*	*Relative difference Baseline vs Shared-effort Imported forest biomass*	*Relative difference Baseline vs Shared-effort Imported energywood from plantations*
RCP6.5	*CFM/SFM*	*noAFM*	2.8E-06	1.8E-06	3.4E-06	2.2E-06	-20%	-27%
RCP6.5	*CFM*	*AFM 12.5%*	2.5E-06	1.7E-06	2.8E-06	2.3E-06	-14%	-38%
RCP6.5	*CFM*	*AFM 25%*	2.4E-06	1.6E-06	2.9E-06	2.2E-06	-19%	-37%
RCP6.5	*CFM*	*AFM 37.5%*	2.6E-06	1.7E-06	2.8E-06	2.5E-06	-7%	-45%
RCP6.5	*CFM*	*AFM 50%*	2.8E-06	1.7E-06	2.9E-06	2.3E-06	-7%	-36%
RCP6.5	*SFM*	*AFM 12.5%*	2.5E-06	1.6E-06	2.9E-06	2.4E-06	-16%	-47%
RCP6.5	*SFM*	*AFM 25%*	3.1E-06	1.7E-06	2.8E-06	2.3E-06	10%	-40%
RCP6.5	*SFM*	*AFM 37.5%*	3.3E-06	1.6E-06	3.4E-06	1.9E-06	-4%	-17%
RCP6.5	*SFM*	*AFM 50%*	3.6E-06	1.6E-06	3.5E-06	1.8E-06	1%	-12%
RCP2.6	*CFM/SFM*	*noAFM*	3.5E-06	2.0E-06	2.9E-06	1.9E-06	18%	4%
RCP2.6	*CFM*	*AFM 12.5%*	3.3E-06	2.0E-06	2.6E-06	2.0E-06	22%	2%
RCP2.6	*CFM*	*AFM 25%*	3.1E-06	2.0E-06	2.5E-06	1.9E-06	21%	2%
RCP2.6	*CFM*	*AFM 37.5%*	3.4E-06	2.0E-06	2.5E-06	1.9E-06	26%	1%
RCP2.6	*CFM*	*AFM 50%*	3.3E-06	1.9E-06	2.5E-06	1.9E-06	23%	4%
RCP2.6	*SFM*	*AFM 12.5%*	3.3E-06	2.0E-06	2.7E-06	1.9E-06	18%	3%
RCP2.6	*SFM*	*AFM 25%*	3.7E-06	1.9E-06	2.9E-06	1.9E-06	22%	2%
RCP2.6	*SFM*	*AFM 37.5%*	3.5E-06	1.9E-06	3.9E-06	1.7E-06	-9%	9%
RCP2.6	*SFM*	*AFM 50%*	3.5E-06	1.9E-06	3.6E-06	1.7E-06	-3%	10%

S16 Spatial distribution of impacts for the different species groups

a) plants

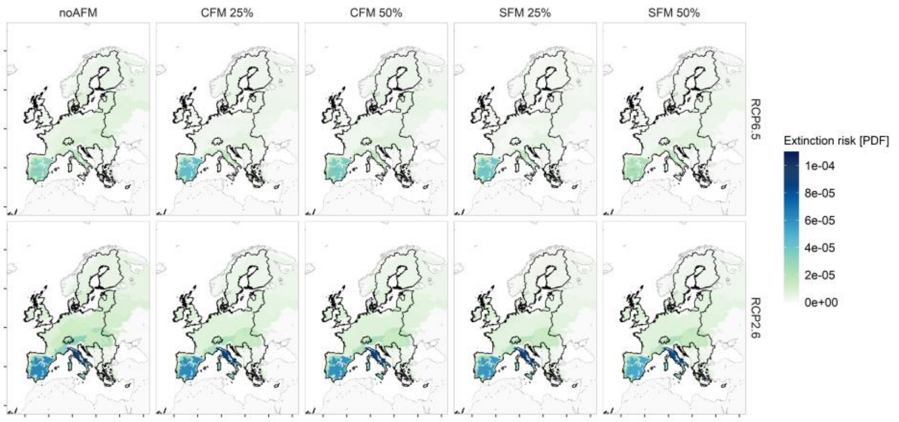

b) birds

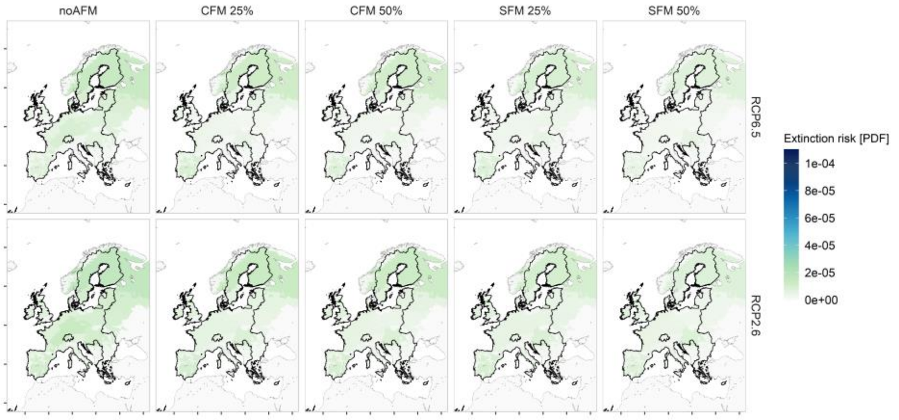

c) mammals

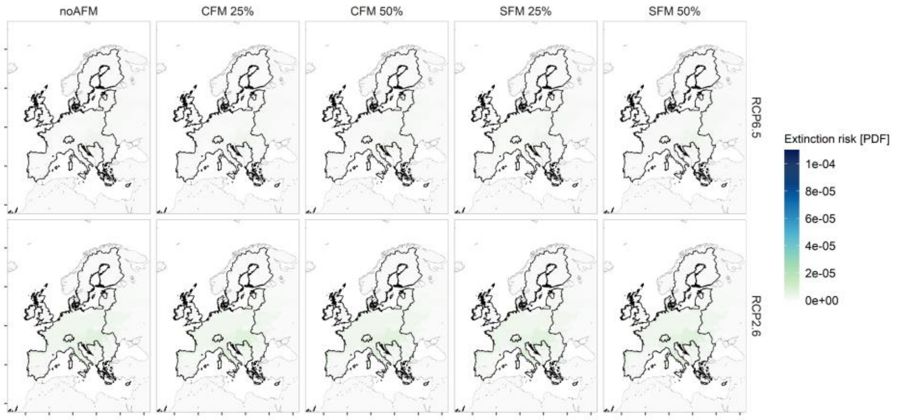

Figure S 16.1 *Baseline scenario - Spatial distribution of the species extinction risk caused by internal EU28 wood and lignocellulosic energy crops production for domestic consumption per species group at ecoregion resolution: a) plants, b) birds and c) mammals (the black line is the EU28 border).*

a) plants

b) birds

c) mammals

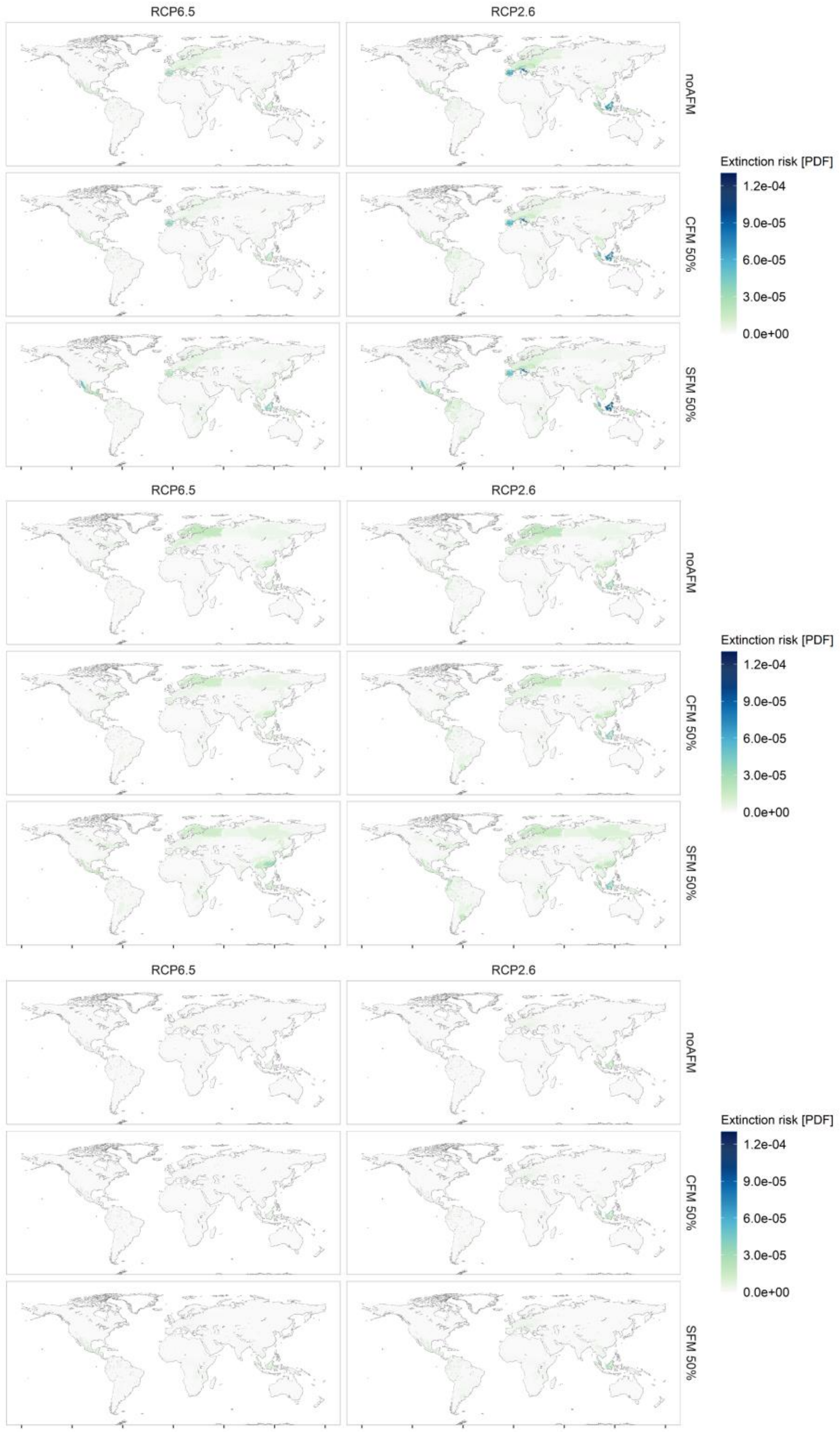

Figure S16.2 *Baseline scenario - Spatial distribution of the species extinction risk caused by EU28 demand of wood and lignocellulosic energy crops per species group at ecoregion resolution: d) plants, e) birds and f) mammals.*

S17 Spatial distribution of the species extinction risk

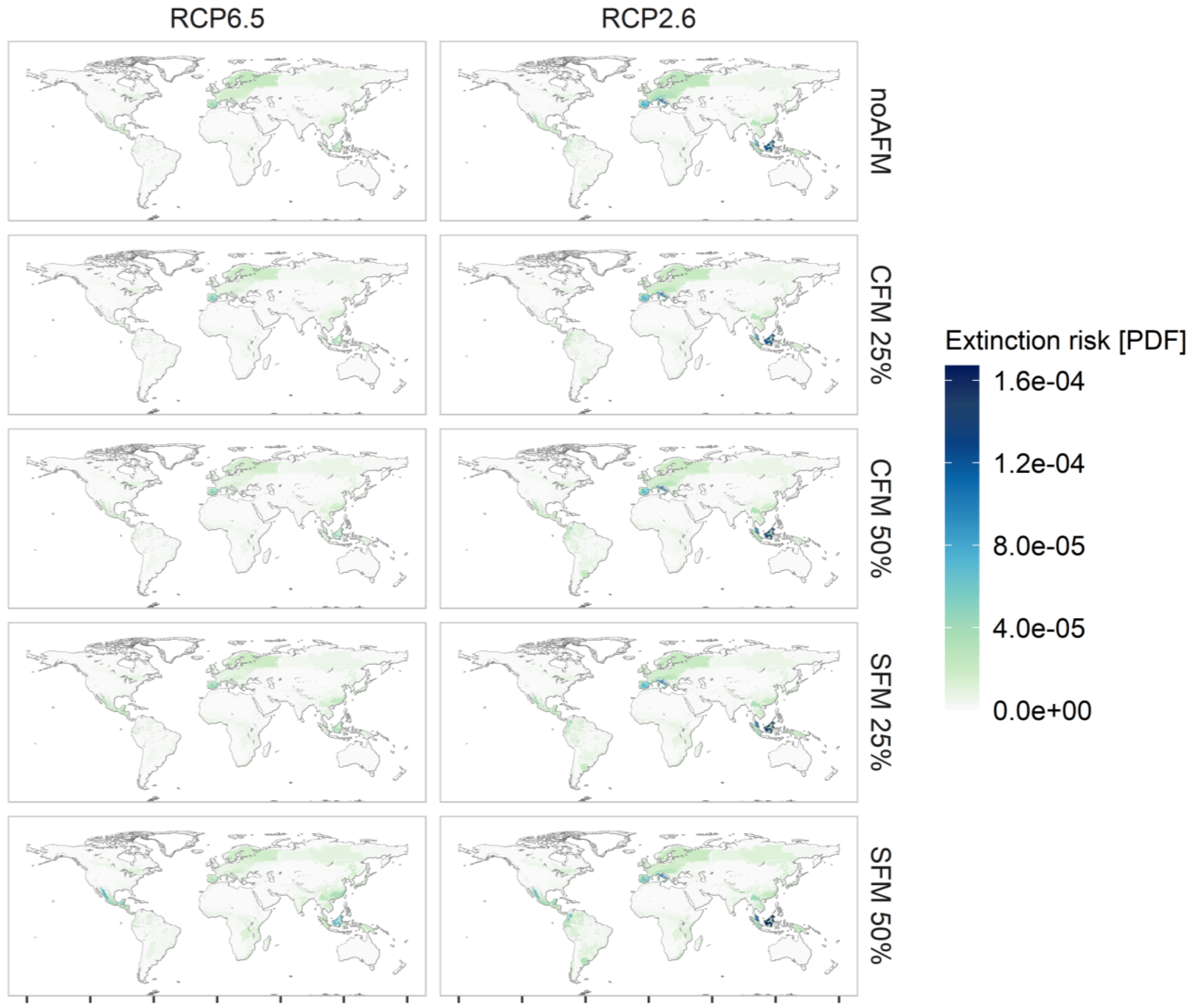

Figure S 17.1 *Baseline scenario - Spatial distribution of species extinction risk in 2100 caused by the EU28 wood and lignocellulosic energy crops demand at ecoregion resolution under the two climate scenarios RCP6.5 and RCP2.6 and multiple alternative forest management scenarios.*

S18 Impacts on the species extinction risk per unit of volume for harvested forest product

Imports gave a high contribution to EU28 forest biomass footprint and, for better disclosing their effect, the impacts per unit of wood product connected to the AFM scenarios under the Baseline scenario were investigated. Data on production, and consequently on species extinction risk per volume unit, refers only to forest practices and land use types modelled in the EU-module (Clear cut, Selection System, Retention, EU28 Energy crops converted from other land use types, imported energy Plantations), therefore, remaining areas allocated to intensive or low-intensity forestry (as described in sec. 2.2 of the main text) were excluded.

Focusing on 2100 (Table S 18.1, Figure S18.1 and 18.2), species loss per Mm^3 of wood harvested in the EU28 is much higher for wood headed to exports than for the one satisfying the internal demand: the latter is partially covered by lands with close-to-nature forest managements like Selection and Retention which have median response ratios equal or very close to one for the species groups assessed. On the contrary, in the Baseline scenario AFMs did not apply to forests used for export, because these were predicted to be intensively managed, according to economic marginal convenience assumed in the land-use model (for a comparison between the PDF/Mm^3 of the Baseline and the Shared-effort scenarios, see Table 15.1).

Even though, as described in the main text, most of the wood is sourced from regions which are not particularly vulnerable (e.g., Canada or USA), 14% of land embodied in wood products was projected to be sourced from tropical regions, which are extremely vulnerable and caused the impacts to reach such high values.

Similarly, the location of the impacts was the reason for a higher species loss per unit of wood due to imported energy plantations when compared to EU28 internal energy crops.

Table S 18.1 *Species extinciton riskper millions of cubic meters (PDF * year /Mm3) of wood harvested under the Baseline scenario within Europe and in areas from which EU28 imports woody biomass in 2100.*

Climate scenario	*Forest use scenario*	*Management scenario*	*EU28 internal forests (no exports)*	*Imported forest biomass*	*EU28 internal energy crops*	*Imported energywood from plantations*
RCP6.5	*CFM/SFM*	*noAFM*	4.00E-07	2.80E-06	6.10E-07	1.80E-06
RCP6.5	*CFM*	*AFM 12.5%*	3.10E-07	2.50E-06	6.20E-07	1.70E-06
RCP6.5	*CFM*	*AFM 25%*	3.10E-07	2.40E-06	6.20E-07	1.60E-06
RCP6.5	*CFM*	*AFM 37.5%*	3.00E-07	2.60E-06	6.10E-07	1.70E-06
RCP6.5	*CFM*	*AFM 50%*	2.90E-07	2.80E-06	5.90E-07	1.70E-06
RCP6.5	*SFM*	*AFM 12.5%*	3.50E-07	2.50E-06	6.10E-07	1.60E-06
RCP6.5	*SFM*	*AFM 25%*	3.60E-07	3.10E-06	5.60E-07	1.70E-06
RCP6.5	*SFM*	*AFM 37.5%*	3.90E-07	3.30E-06	5.60E-07	1.60E-06
RCP6.5	*SFM*	*AFM 50%*	5.40E-07	3.60E-06	5.30E-07	1.60E-06
RCP2.6	*CFM/SFM*	*noAFM*	3.90E-07	3.50E-06	5.10E-07	2.00E-06
RCP2.6	*CFM*	*AFM 12.5%*	2.80E-07	3.30E-06	5.00E-07	2.00E-06
RCP2.6	*CFM*	*AFM 25%*	2.80E-07	3.10E-06	5.00E-07	2.00E-06
RCP2.6	*CFM*	*AFM 37.5%*	2.80E-07	3.40E-06	5.00E-07	2.00E-06
RCP2.6	*CFM*	*AFM 50%*	2.80E-07	3.30E-06	5.10E-07	1.90E-06
RCP2.6	*SFM*	*AFM 12.5%*	3.00E-07	3.30E-06	5.00E-07	2.00E-06
RCP2.6	*SFM*	*AFM 25%*	3.40E-07	3.70E-06	5.10E-07	1.90E-06
RCP2.6	*SFM*	*AFM 37.5%*	4.10E-07	3.50E-06	5.00E-07	1.90E-06
RCP2.6	*SFM*	*AFM 50%*	5.70E-07	3.50E-06	5.00E-07	1.90E-06

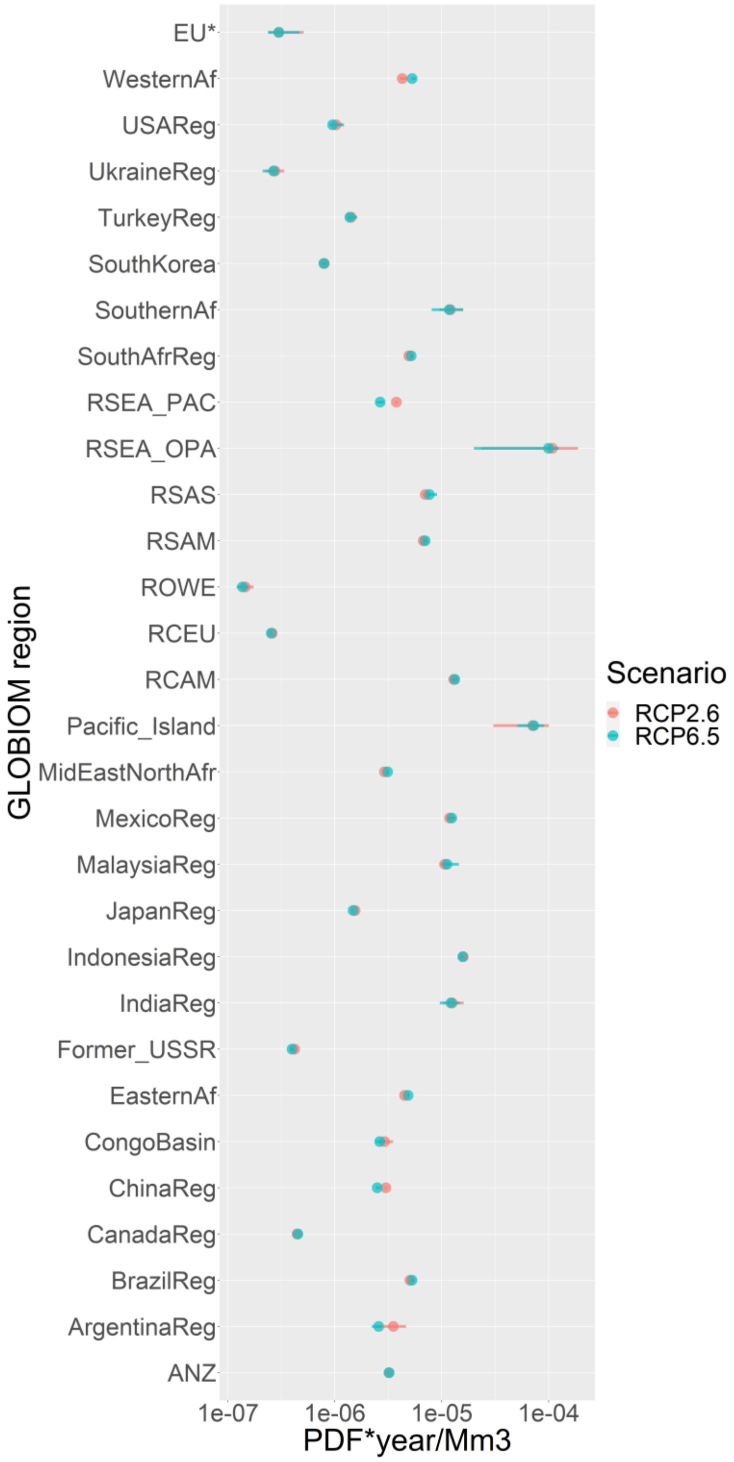

Figure S 18.1 *Exinction risk per millions of cubic meters of wood harvested in 2100 in the GLOBIOM regions under the Baseline scenario caused by EU28 forest biomass imports for energy-unrelated purposes. The points correspond to the average global PDF*year/Mm3 of the forest management scenarios in each climate scenario, whereas the lines indicate the minimum and the maximum. The countries which belong to each GLOBIOM region are listed in Table S4.1. * The EU values are added as reference and correspond to the global PDF*year/Mm3 of EU28 internal production for domestic consumption (no exports).*

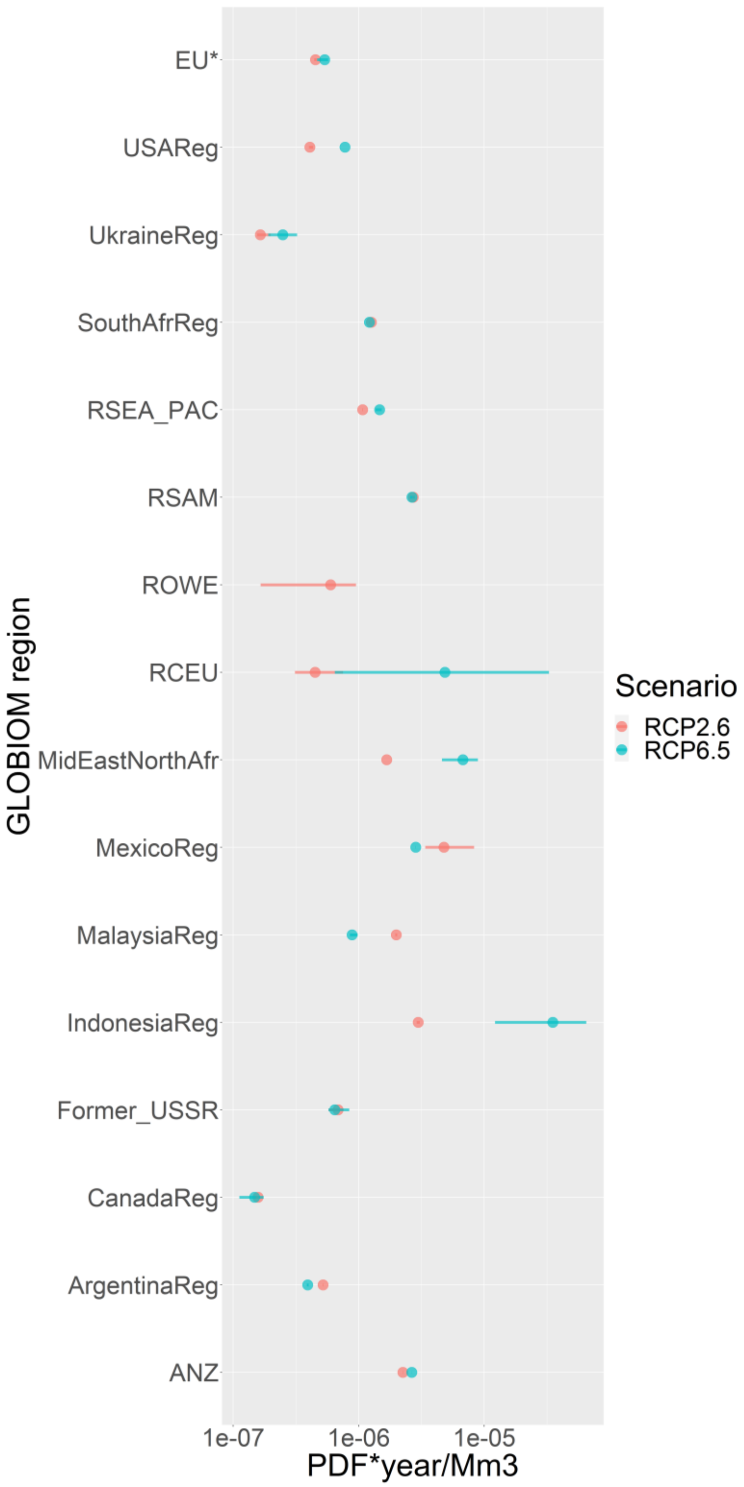

Figure S 16.2 *Species extinction risk per millions of cubic meters of wood harvested in 2100 in the GLOBIOM regions under the Baseline scenario caused by EU28 imports of energywood from plantations. The points correspond to the average PDF*year/Mm3 of the forest management scenarios in each climate scenario, whereas the lines indicate the minimum and the maximum. The countries which belong to each GLOBIOM region are listed in Table S4.2. * The EU values are added as reference and correspond to the PDF*year/Mm3 of EU28 internal production for domestic consumption (no exports).*

S19 Difference and ratio compared to noAFM

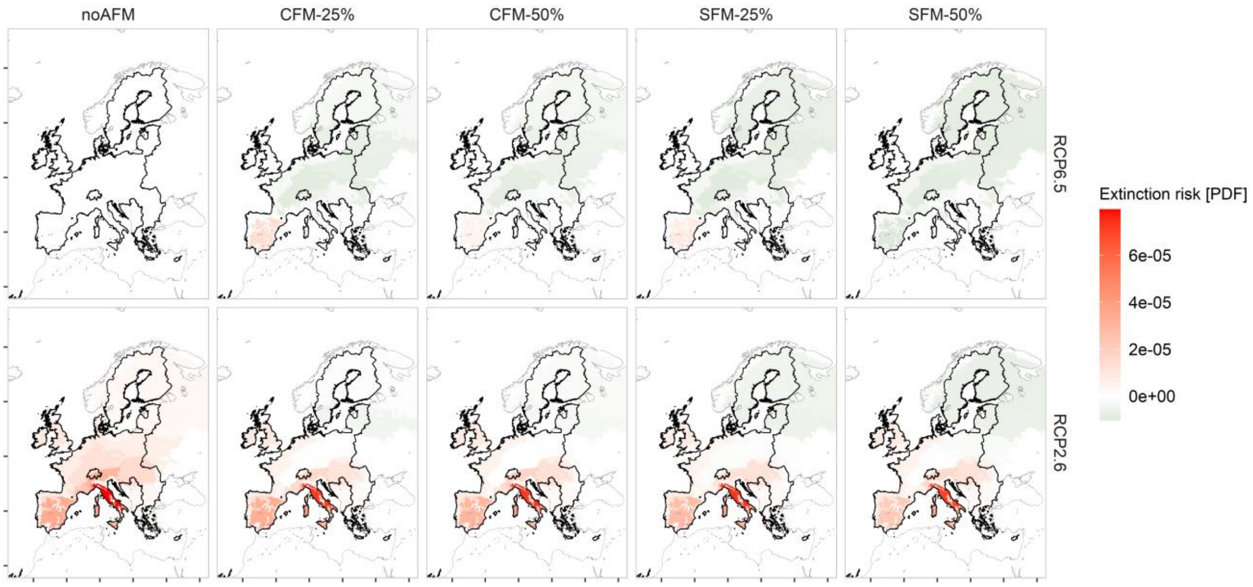

Figure S 19.1 *Baseline scenario - Spatial distribution of the difference in 2100 between species extinction risk of the scenarios under AFMs and species extinction risk in noAFM scenario (the black line is the EU28 border) due to EU28 internal forest management and lignocellulosic energy crops for domestic use.*

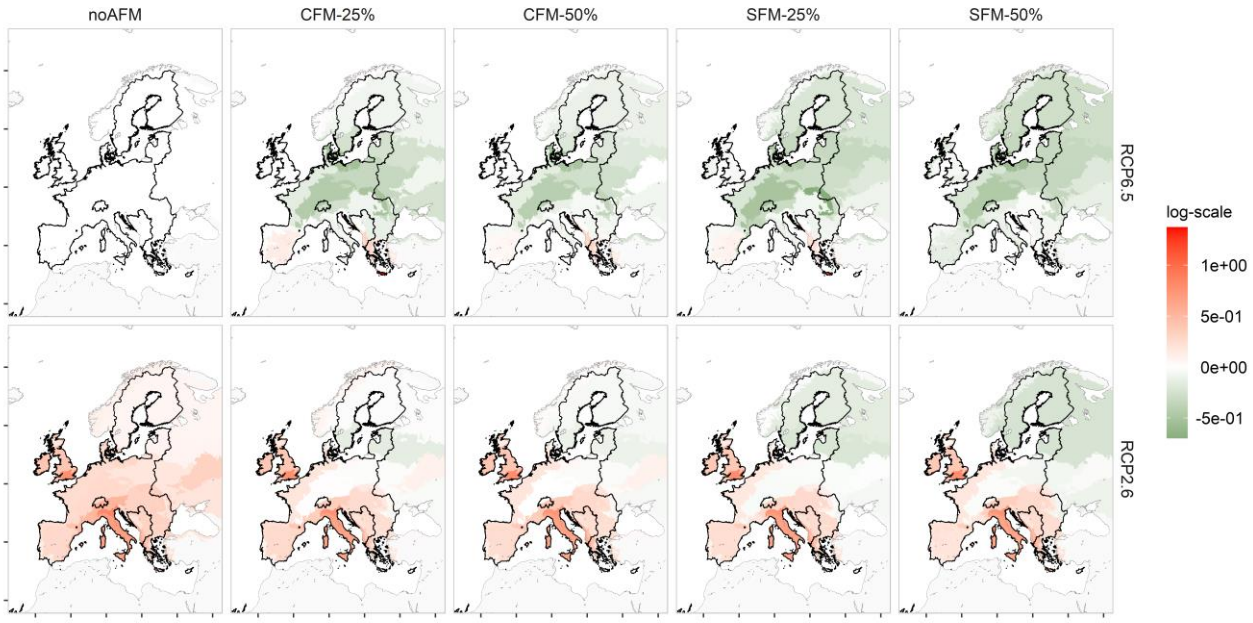

Figure S 19.2 *Baseline scenario - Spatial distribution of the ratio in 2100 between species extinction risk of the scenarios under AFMs and species extinction risk in noAFM (the black line is the EU28 border) due to EU28 internal forest management and lignocellulosic energy crops for domestic use.*

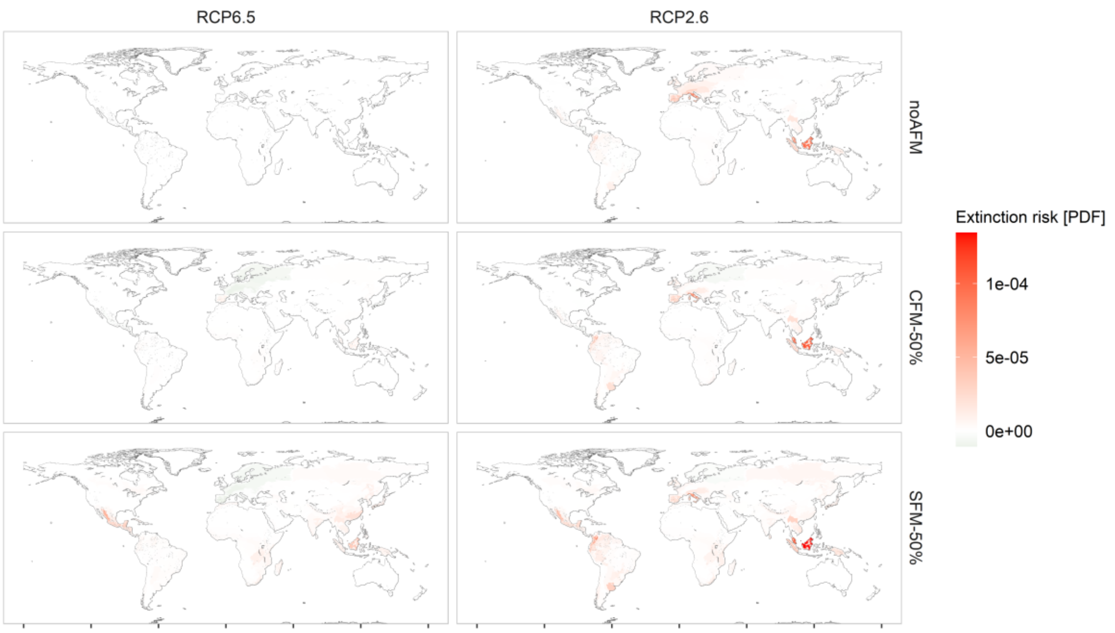

Figure S 19.3 *Baseline scenario - Spatial distribution of the difference in 2100 between species extinction risk of the scenarios under AFMs and species extinction risk in noAFM caused by the EU28 wood and lignocellulosic energy crops demand at ecoregion resolution.*

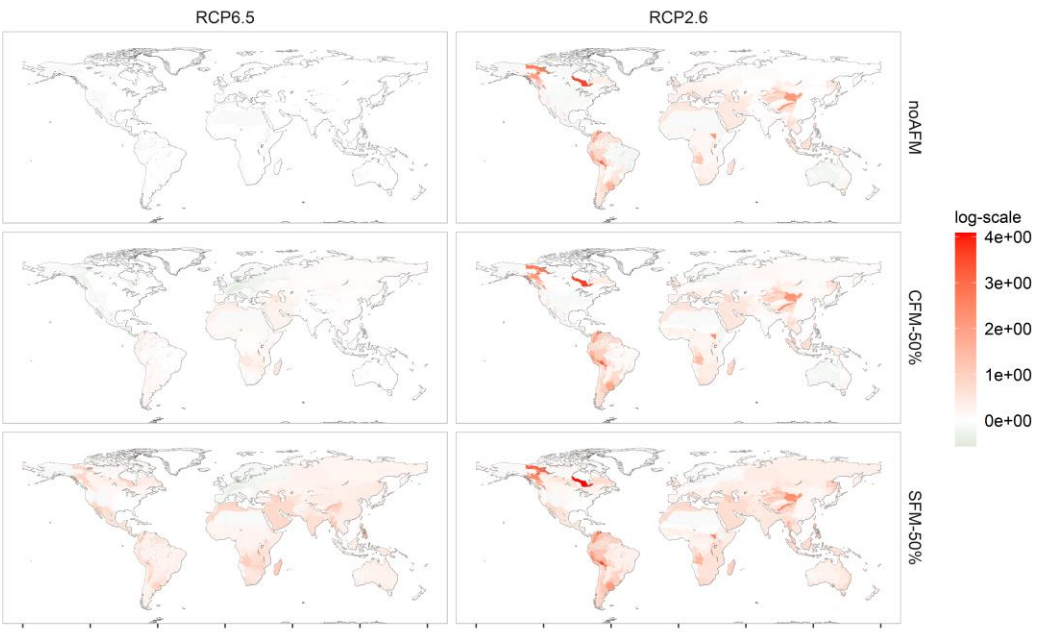

Figure S 19.4 *Baseline scenario - Spatial distribution of the ratio 2100 between species extinction risk of the scenarios under AFMs and species extinction risk in noAFM caused by the EU28 wood and lignocellulosic energy crops demand at ecoregion resolution.*

S20 Spatial distribution of impacts in the Shared-effort scenario

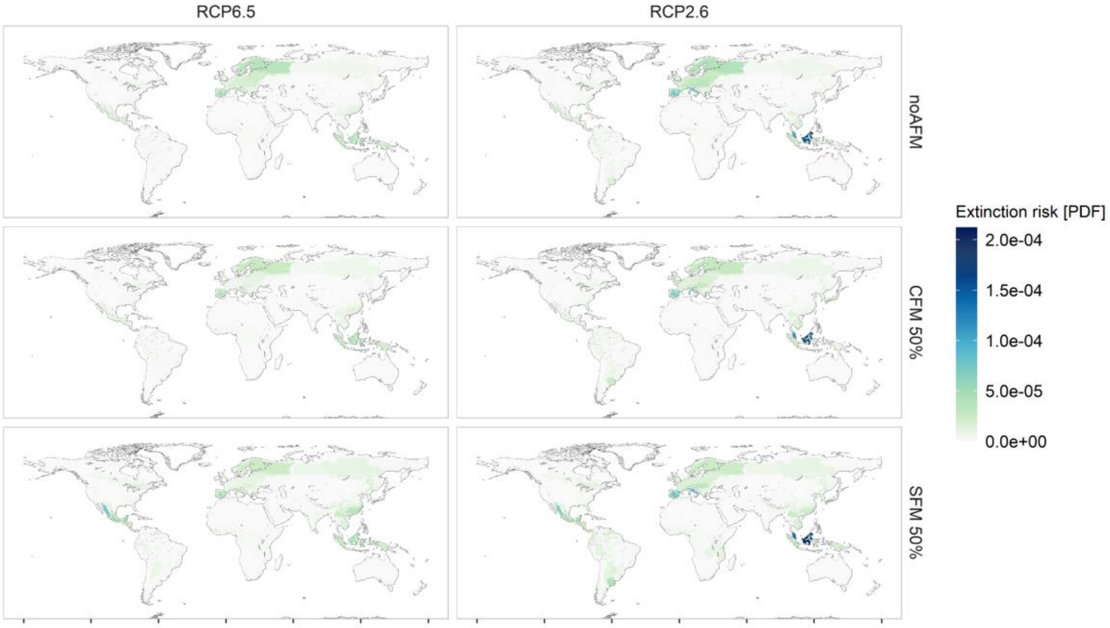

Figure 20.1 *Shared-effort scenario - Spatial distribution of species extinction risk in 2100 caused by the EU28 wood and lignocellulosic energy crops demand at ecoregion resolution under the two climate scenarios RCP6.5 and RCP2.6 and the most extreme alternative forest management scenarios, where half of EU28 forestland is converted to closer-to-nature practices or to set-asides.*

S21 Areas converted to lignocellulosic energy crops and energy plantations

Table S 21.1 *Baseline scenario - Land converted (Mha) from 2020 to 2100 to lignocellulosic energy crops within the EU28 and to energy plantations outside the EU28 to meet the EU28 demand of non-agricultural biomass for energy production.*

Climate scenario	*Forest use scenario*	*Manage-ment scenario*	*Croplands converted to energy plantations (outside the EU28)*	*Grasslands converted to energy plantations (outside the EU28)*	*Natural lands converted to energy plantations (outside the EU28)*	*Croplands converted to lignocel-lulosic energy crops (within the EU28)*	*Grasslands converted to lignocellu-losic energy crops (within the EU28)*	*Natural lands converted to lignocellulosi c energy crops (within the EU28)*
RCP6.5	*CFM/SFM*	*noAFM*	0.57	0.21	0.30	2.31	0.95	1.47
RCP6.5	*CFM*	*AFM 12.5%*	0.67	0.28	0.36	2.12	0.86	1.37
RCP6.5	*CFM*	*AFM 25%*	0.76	0.29	0.37	2.20	0.92	1.42
RCP6.5	*CFM*	*AFM 37.5%*	0.59	0.23	0.30	2.45	0.98	1.45
RCP6.5	*CFM*	*AFM 50%*	0.83	0.37	0.46	2.72	1.00	1.50
RCP6.5	*SFM*	*AFM 12.5%*	0.78	0.32	0.42	2.25	0.94	1.42
RCP6.5	*SFM*	*AFM 25%*	0.89	0.38	0.48	2.77	1.14	1.58
RCP6.5	*SFM*	*AFM 37.5%*	1.18	0.45	0.55	3.22	1.26	1.69
RCP6.5	*SFM*	*AFM 50%*	2.03	0.84	1.06	3.83	1.39	1.76
RCP2.6	*CFM/SFM*	*noAFM*	4.77	0.97	2.09	7.58	2.78	3.50
RCP2.6	*CFM*	*AFM 12.5%*	4.83	1.03	2.13	7.54	2.74	3.49
RCP2.6	*CFM*	*AFM 25%*	5.49	1.18	2.37	7.73	2.82	3.60
RCP2.6	*CFM*	*AFM 37.5%*	5.90	1.31	2.55	8.29	2.95	3.70
RCP2.6	*CFM*	*AFM 50%*	6.79	1.55	2.92	8.23	2.97	3.76
RCP2.6	*SFM*	*AFM 12.5%*	5.48	1.17	2.40	7.94	2.87	3.61
RCP2.6	*SFM*	*AFM 25%*	6.78	1.51	2.93	8.46	3.04	3.81
RCP2.6	*SFM*	*AFM 37.5%*	8.17	2.10	3.70	8.97	3.28	3.95
RCP2.6	*SFM*	*AFM 50%*	9.16	2.47	4.27	9.45	3.43	4.09

References

(1) Chaudhary, A.; Burivalova, Z.; Koh, L. P.; Hellweg, S. Impact of Forest Management on Species Richness: Global Meta-Analysis and Economic Trade-Offs. *Sci. Rep.* **2016**, *6*, 1–10. https://doi.org/10.1038/srep23954.

(2) FAO. FAOSTAT Database https://www.fao.org/faostat (accessed Jan 10, 2020).

(3) Nachtergaele, F.; Petri, M. *Mapping Land Use Systems at Global and Regional Scales for Land Degradation Assessment Analysis. LADA Technical Report Number 8, Version 1.1*; 2008.

(4) Ellis, E. C.; Ramankutty, N. Putting People in the Map: Anthropogenic Biomes of the World. *Front. Ecol. Environ.* **2008**, *6* (8), 439–447. https://doi.org/10.1890/070062.

(5) Chaudhary, A.; Verones, F.; De Baan, L.; Hellweg, S. Quantifying Land Use Impacts on Biodiversity: Combining Species-Area Models and Vulnerability Indicators. *Environ. Sci. Technol.* **2015**, *49* (16), 9987–9995. https://doi.org/10.1021/acs.est.5b02507.

(6) Chaudhary, A.; Verones, F.; De Baan, L.; Pfister, S.; Hellweg, S. Chapter 11 - Land Stress: Potential Species Loss Form Land Use. *LC-IMPACT Version 1.0, www.lc-impact.eu.* **2016**.

(7) Curran, M.; Hellweg, S.; Beck, J. Is There Any Empirical Support for Biodiversity Offset Policy? *Ecol. Appl.* **2014**, *24* (4), 617–632. https://doi.org/10.1890/13-0243.1.

(8) Pezzati, L.; Verones, F.; Curran, M.; Baustert, P.; Hellweg, S. Biodiversity Recovery and Transformation Impacts for Wetland Biodiversity. *Environ. Sci. Technol.* **2018**, *52* (15), 8479–8487. https://doi.org/10.1021/acs.est.8b01501.

(9) Pereira, H. M.; Ziv, G.; Miranda, M. Countryside Species-Area Relationship as a Valid Alternative to the Matrix-Calibrated Species-Area Model. *Conserv. Biol.* **2014**, *28* (3), 874–876. https://doi.org/10.1111/cobi.12289.

(10) Lindenmayer, D. B.; Fischer, J. How Landscape Change Affects Organisms: A Conceptual Framework. In *Habitat fragmentation and landscape change: An ecological and*

conservation synthesis; Island Press, 2006; pp 26–38.

(11) Forman, R. T. *Land Mosaics: The Ecology of Landscapes and Regions*, 2014th ed.; Springer: New York, 1995.

(12) Frischknecht, R.; Jolliet, O. *Global Guidance for Life Cycle Impact Assessment Indicators Volume 1*; 2016; Vol. 1.

(13) Lauri, P.; Forsell, N.; Di Fulvio, F.; Snäll, T.; Havlik, P. Material Substitution between Coniferous, Non-Coniferous and Recycled Biomass – Impacts on Forest Industry Raw Material Use and Regional Competitiveness. *For. Policy Econ.* **2021**, *132* (February), 102588. https://doi.org/10.1016/j.forpol.2021.102588.

(14) Havlík, P.; Schneider, U. A.; Schmid, E.; Böttcher, H.; Fritz, S.; Skalský, R.; Aoki, K.; Cara, S. De; Kindermann, G.; Kraxner, F.; Leduc, S.; McCallum, I.; Mosnier, A.; Sauer, T.; Obersteiner, M. Global Land-Use Implications of First and Second Generation Biofuel Targets. *Energy Policy* **2011**, *39* (10), 5690–5702. https://doi.org/10.1016/j.enpol.2010.03.030.

(15) Havlík, P.; Valin, H.; Herrero, M.; Obersteiner, M.; Schmid, E.; Rufino, M. C.; Mosnier, A.; Thornton, P. K.; Böttcher, H.; Conant, R. T.; Frank, S.; Fritz, S.; Fuss, S.; Kraxner, F.; Notenbaert, A. Climate Change Mitigation through Livestock System Transitions. *Proc. Natl. Acad. Sci. U. S. A.* **2014**, *111* (10), 3709–3714. https://doi.org/10.1073/pnas.1308044111.

(16) Drakare, S.; Lennon, J. J.; Hillebrand, H. The Imprint of the Geographical, Evolutionary and Ecological Context on Species-Area Relationships. *Ecol. Lett.* **2006**, *9* (2), 215–227. https://doi.org/10.1111/j.1461-0248.2005.00848.x.

(17) IUCN (International Union for Conservation of Nature and Natural Resources). IUCN Red List http://www.iucnredlist.org/technical-documents/spatial-data.

(18) Verones, F.; Pfister, S.; van Zelm, R.; Hellweg, S. Biodiversity Impacts from Water Consumption on a Global Scale for Use in Life Cycle Assessment. *Int. J. Life Cycle Assess.*

2017, *22* (8), 1247–1256. https://doi.org/10.1007/s11367-016-1236-0.

(19) De Baan, L.; Mutel, C. L.; Curran, M.; Hellweg, S.; Koellner, T. Land Use in Life Cycle Assessment: Global Characterization Factors Based on Regional and Global Potential Species Extinction. *Environ. Sci. Technol.* **2013**, *47* (16), 9281–9290. https://doi.org/10.1021/es400592q.

(20) Chaudhary, A.; Brooks, T. M. Land Use Intensity-Specific Global Characterization Factors to Assess Product Biodiversity Footprints. *Environ. Sci. Technol.* **2018**, *52* (9), 5094–5104. https://doi.org/10.1021/acs.est.7b05570.
